# A Machine Learning Approach for Identifying Novel Cell Type–Specific Transcriptional Regulators of Myogenesis

**DOI:** 10.1371/journal.pgen.1002531

**Published:** 2012-03-08

**Authors:** Brian W. Busser, Leila Taher, Yongsok Kim, Terese Tansey, Molly J. Bloom, Ivan Ovcharenko, Alan M. Michelson

**Affiliations:** 1 Laboratory of Developmental Systems Biology, National Heart, Lung, and Blood Institute, National Institutes of Health, Bethesda, Maryland, United States of America; 2 Computational Biology Branch, National Center for Biotechnology Information, National Library of Medicine, National Institutes of Health, Bethesda, Maryland, United States of America; University of California San Diego, United States of America

## Abstract

Transcriptional enhancers integrate the contributions of multiple classes of transcription factors (TFs) to orchestrate the myriad spatio-temporal gene expression programs that occur during development. A molecular understanding of enhancers with similar activities requires the identification of both their unique and their shared sequence features. To address this problem, we combined phylogenetic profiling with a DNA–based enhancer sequence classifier that analyzes the TF binding sites (TFBSs) governing the transcription of a co-expressed gene set. We first assembled a small number of enhancers that are active in *Drosophila melanogaster* muscle founder cells (FCs) and other mesodermal cell types. Using phylogenetic profiling, we increased the number of enhancers by incorporating orthologous but divergent sequences from other *Drosophila* species. Functional assays revealed that the diverged enhancer orthologs were active in largely similar patterns as their *D. melanogaster* counterparts, although there was extensive evolutionary shuffling of known TFBSs. We then built and trained a classifier using this enhancer set and identified additional related enhancers based on the presence or absence of known and putative TFBSs. Predicted FC enhancers were over-represented in proximity to known FC genes; and many of the TFBSs learned by the classifier were found to be critical for enhancer activity, including POU homeodomain, Myb, Ets, Forkhead, and T-box motifs. Empirical testing also revealed that the T-box TF encoded by *org-1* is a previously uncharacterized regulator of muscle cell identity. Finally, we found extensive diversity in the composition of TFBSs within known FC enhancers, suggesting that motif combinatorics plays an essential role in the cellular specificity exhibited by such enhancers. In summary, machine learning combined with evolutionary sequence analysis is useful for recognizing novel TFBSs and for facilitating the identification of cognate TFs that coordinate cell type–specific developmental gene expression patterns.

## Author Summary

The development of multicellular organisms requires the formation of a diversity of cell types. Each cell has a unique genetic program that is orchestrated by regulatory sequences called enhancers, comprising multiple short DNA sequences that bind distinct transcription factors. Understanding developmental regulatory networks requires knowledge of the sequence features of functionally related enhancers. We developed an integrated evolutionary and computational approach for deciphering enhancer regulatory codes and applied this method to discover new components of the transcriptional network controlling muscle development in the fruit fly, *Drosophila melanogaster*. Our method involves assembling known muscle enhancers, expanding this set with evolutionarily conserved sequences, computationally classifying these enhancers based on their shared sequence features, and scanning the entire *Drosophila* genome to predict additional related enhancers. Using this approach, we created a map of 5,500 putative muscle enhancers, identified candidate transcription factors to which they bind, observed a strong correlation between mapped enhancers and muscle gene expression, and uncovered extensive heterogeneity among combinations of transcription factor binding sites in validated muscle enhancers, a feature that may contribute to the individual cellular specificities of these regulatory elements. Our strategy can readily be generalized to study transcriptional networks in other organisms and developmental contexts.

## Introduction

Complex spatio-temporal gene expression programs guide the progressive determination of pluripotent cells allowing cell fates to become sequentially restricted during embryonic development. These transitions in cell fate are encoded in the genome by *cis* regulatory DNA sequences such as transcriptional enhancers. Enhancers respond to the combinatorial input of tissue-specific, cell-specific, ubiquitously-expressed and signal-activated transcription factors (TFs) that collectively control gene expression in the appropriate spatial and temporal patterns [1], [2].

In recent years, we and others have shown that computational approaches can be used to predict enhancers of a given type with reasonable accuracy when prior knowledge exists of the TFs and their binding sites that contribute to the activity of this enhancer class [3]–[5]. However, this approach is limited when the identities and the binding site sequences of co-regulatory TFs are not known. To circumvent this problem, several groups have identified enhancers based on the presence of shared sequence features without the necessity of knowing the co-regulating TFs or their binding motifs [6]–[12]. These enhancer modeling approaches generally take advantage of two data sources: (1) the non-coding sequences surrounding the members of a gene set of interest, or a set of previously validated enhancers associated with such genes; and (2) previously described sequence motifs from transcription factor binding site (TFBS) libraries and/or *de novo* motif discovery. In this way, previously described or candidate motifs and/or word profiles can be used to ascertain a training set of enhancers, with the resulting model being used in a genome-wide scan to predict similar enhancers. The enhancer model is validated by testing the activity of these predictions in transgenic reporter assays [7], [13]. A particular transcriptional regulatory model can also be validated by assaying the functionality of the motifs that are found to be relevant for making predictions, and subsequently by identifying the DNA binding proteins that target these sequences.

The majority of the studies showing the utility of enhancer modeling have focused on regulatory sequences involved in segmentation of the *Drosophila* blastoderm embryo [11], [13]–[15]. Furthermore, we have recently demonstrated that enhancer modeling can be used to reveal the enhancers and constituent sequence motifs involved in human heart development [7]. Surprisingly, recently predicted blastoderm segmentation enhancers were often active in other tissues and developmental stages [13], whereas the validation rate for predicted human heart enhancers was much higher [7]. These differences in success rates could reflect methodology or might reflect the composition of the training set of sequences. In support of the latter possibility, there are sequence features unique to the blastoderm segmentation enhancers which might limit their amenability to this approach [16].

The development of the *Drosophila* larval somatic, visceral and heart muscles from mesodermal progenitors requires the coordinated input of multiple different regulators, including the intrinsic TFs Twist (Twi), Tinman (Tin) and Mef2 [17], [18], and the intercellular signaling pathways mediated by the epidermal growth factor, fibroblast growth factor, Wnt, hedgehog and bone morphogenetic proteins (BMPs) [18]–[20]. These tissue-specific and downstream signal-activated TFs are highly conserved in sequence and function from *Drosophila* to vertebrates [21]. Although these factors function in various combinations to confer general and subtype properties on differentiating mesodermal cells, they also have pleiotropic effects in development such that additional factors are required to specify individual cellular identities. For example, the *Drosophila* larval somatic muscles are multinucleated myotubes each having unique properties that include their size, shape, orientation, epidermal attachments and innervation [18], [19]. The formation of each myotube is initiated by a single muscle founder cell (FC) whose fate prefigures that of the corresponding muscle and is controlled by the combinatorial activities of muscle FC identity TFs [18], [19]. FCs fuse with a more homogeneous population of neighboring muscle cells termed fusion-competent myoblasts (FCMs) to form muscle precursors [18], [19]. The complexity of FC genetic programs [22] necessitates that a large number of identity TFs be involved in their specification, yet only a small number of such factors are known [20], few direct targets of these factors have been characterized, and little information is available about the combinatorial control of FC enhancers by TFs of different classes.

Here we applied evolutionary and machine learning approaches to model *Drosophila* mesodermal enhancers having FC activities in order to uncover the motifs that orchestrate gene expression at the level of individual cells, to generate testable hypotheses about the nature of the corresponding FC identity TFs, and to gain insights into the combinations of TFs that contribute to individual FC enhancer specificities. The coordinated input of tissue-specific and signal-activated TFs, combined with the discrete identities of individual FCs, suggests that the regulatory network specifying distinct FC genetic programs is likely to share some common features while differing substantially with respect to others. Furthermore, a series of studies by Erives and colleagues has shown that a family of non-homologous enhancers is characterized by a discrete regulatory signature [23]–[25] in spite of the inherent complexity of isolated enhancers [26], [27]. Taken together, this information suggests that the FC regulatory network should be amenable to an enhancer modeling approach.

To address this problem, we first compiled a small set of enhancers with activity in FCs. To overcome issues associated with small sample sizes, and to increase the diversity of sequences with similar functions, we extended this set by adding orthologs derived from other *Drosophila* species. *In vivo* testing revealed that these orthologous sequences are functional FC enhancers in spite of having extensive reorganization of their DNA sequences. We show that increasing the training set through the addition of orthologous sequences improves the performance of our enhancer prediction model. By training on this extended set of enhancers, we were able to computationally predict functionally relevant TFBSs and enhancers for the FC gene set. When the resulting classifier was run genome-wide to search for new *D. melanogaster* FC enhancers, we identified 5,500 high-scoring predictions at a false-positive rate (FPR) of 5%. Moreover, these predicted enhancers were significantly enriched in the noncoding regions associated with known FC genes. While many of the TFBSs learned by the classifier are known to regulate the transcription of muscle FC genes, our classifier predicted additional motifs which have not previously been identified as contributing to FC enhancer activities. Site-directed mutagenesis of five newly discovered motifs in previously characterized FC enhancers demonstrated the critical role played by these TFBSs in supporting full enhancer activity. These validated motifs also suggest plausible candidate TFs acting in the myogenic regulatory network. In one such case—that of the T-box protein encoded by *optomotor-blind-related-gene-1* (*org-1*)—we were able to use loss- and gain-of-function genetic perturbations to establish that this TF functions as a regulator of muscle identity. Furthermore, an analysis of the TFBS compositions of all known FC enhancers revealed an unanticipated complexity in the combinations of TFs that contribute to the unique specificities of individual regulatory elements, a finding that provides a molecular explanation for the well-known diversity of muscle cell identities and their associated gene expression programs.

## Results

**Figure 1:**
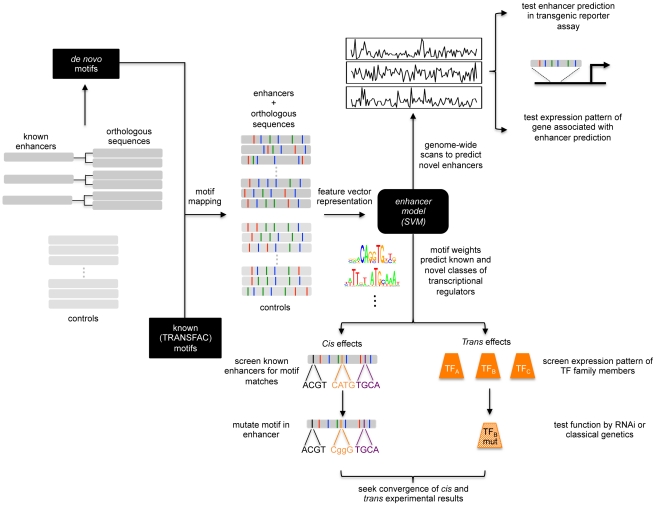
Schematic of enhancer classification beginning with a small training set. A small set of known enhancers active in similar cells is increased by incorporating orthologous sequences. *De novo* and known motifs are mapped onto this training set and a set of control sequences. Feature vectors are used to build an enhancer model based upon the learned motif weighting. This model can be used to scan the genome for similar enhancers as the training set. These predictions can be tested using transgenic reporter assays or analysis of the expression of the associated gene. The motif weighting can likewise be used to identify novel classes of transcriptional regulators. The role of the motifs can be tested in *cis* and the identification of co-regulating TFs can be subsequently tested in *trans*.

Here we utilized phylogenetic profiling and machine learning to decipher the motifs and enhancers that underlie the gene expression patterns of individual muscle FCs, which required an array of computational and experimental tools. This study is composed of 4 main components: (1) compiling a training set of FC enhancers from multiple sources including the literature, testing of additional computational predictions from a previous study [5], increasing the size of the dataset through phylogenetic profiling, including the empirical validation of a subset of those predictions; (2) machine learning on the FC enhancer training set; (3) experimental validation of classifier predictions using transgenic reporter assays and whole embryo *in situ* hybridization with gene-specific probes; and (4) functional examination of sequence features associated with the computational classification to define novel motifs and TFs regulating myogenesis. An overview of the approach utilized in this study is presented in Figure 1. In addition, we used the information derived from the abovementioned studies to examine the distribution of TFBSs across the entire set of known FC enhancers to ascertain the extent to which TF combinatorics contributes to the diversity of FC enhancer activities.

### Building a Training Set of Enhancers That Are Active in Muscle FCs

Previous studies have characterized enhancers for individual FC genes that integrate many of the TFs downstream of the Wnt, Ras/MAPK and BMP signaling pathways, as well as input from the instrinsic TFs Twi and Tin [28]–[31]. However, relatively sparse information is available from these examples to understand the full complexity of the myogenic regulatory network. To begin unraveling the detailed architecture of this network, we previously used expression profiling of various mutants which perturb FC gene expression in a predictable manner to identify hundreds of candidate genes with FC expression patterns [22]. *In situ* hybridization of these candidates led to the validation of 180 FC genes (Table S1). To understand how these FC genes are coordinately regulated, we evaluated potential regulatory codes which were based on combinations of TFBSs found within two previously characterized FC enhancers [28], [29]. These studies revealed that three TFs—Twi, Tin, and Pointed (Pnt), an Ets-domain TF acting downstream of Ras/MAPK signaling—combine to regulate a subset of FC genes (termed C1) that are particularly Ras- and Pnt-responsive [5]. Furthermore, we originally showed that 3 out of 4 genomic regions associated with C1 FC genes that contain clusters of binding sites for Pnt, Twi and Tin are functional FC enhancers when tested in transgenic reporter assays [5]. To extend that study, we have now tested 16 more predicted enhancers associated with C1 FC genes and found that 8 of these are bona fide FC regulatory elements (Figure S1 and Table S1). In contrast, only 2 out of 18 similarly selected candidate regions associated with non-C1 FC genes were validated as FC enhancers, although 4 of these predicted elements were active in other mesodermal tissues (Table S1). Similar to our previous work [5], these enhancers are active in differing subsets of the 30 individual FCs per hemisegment, with the only requirement being activity in one or more FCs. In total, these findings suggest that the transcriptional code governing C1 FC gene expression is missing one or more critical regulatory components, including cell type-specific factors.

Interestingly, the activities of 14 of these 16 FC enhancers are not restricted to FCs but also include other mesodermal and non-mesodermal cell types (Table S1). It is generally believed that an individual enhancer controls a particular spatio-temporal aspect of a gene's total expression pattern, with each enhancer composed of distinct clusters of binding sites for different combinations of TFs [2]. However, we have recently shown that the enhancer for the *Drosophila Nidogen (Ndg)* gene is active at different developmental stages and in multiple cell types (including FCs) due to the binding of multiple cell-specific TFs of the same family ([5] and X. Zhu, S. M. Ahmad, A. Aboukhalil, B. W. Busser, Y. Kim, T. R. Tansey, A. Haimovich, N. Jeffries, M. L. Bulyk, and A. M. Michelson, unpublished data). In this context, it is important to note that in several cases where attempts have been made to separate FC from other sites of mesodermal activity, it has not been possible to identify independent enhancers for the different cell types [5], [28]. Furthermore, a survey of *Drosophila* enhancers shows that the majority are active in multiple cell types ([32] and see Table S2). A similar survey of vertebrate enhancers shows that this diversity of enhancer activities is not a reflection of the relatively compact *Drosophila* genome ([33], [34] and data not shown). Thus, the regulation of some genes occurs through multiple enhancers, with each individual enhancer directing a specific spatio-temporal aspect of a particular gene's expression. In contrast, other genes are regulated by a single enhancer which directs the entirety (or a large fraction) of the spatio-temporal expression pattern of the gene through the combinatorial activities of TFs that themselves have cell type restricted expression. The latter model appears to predominate for *Drosophila* FC enhancers. Despite the potential challenges of machine learning on a set of regulatory sequences having broad expression activities, our goal was to use existing information about FC gene regulation to identify both additional enhancers and novel TFBSs that convey individual FC specificity (Figure 1).

### Sequences Orthologous to Known FC Enhancers Have Similar Regulatory Functions

Combining the aforementioned studies and previously published work, the training set contained a total of 16 FC enhancers [5], [28], [29], [35], [36]. Machine learning approaches require large and representative datasets to learn robust decision rules. Small training sets often lead to over-fitting of such decision rules and, consequently, do not satisfactorily generalize data that vary slightly in their statistical structure. In addition, limited datasets are likely to only partially represent the distribution of all instances of their class. Thus, to accurately learn the TFBSs that are responsible for FC gene regulation, and to reliably predict additional related enhancers, we investigated options to expand the set of training sequences. This goal was accomplished by a phylogenetic profiling approach which integrates orthologous sequences from the genomes of the 11 other fully sequenced *Drosophila* species, mosquito, honeybee and red flour beetle by searching for regions displaying at least 50% but less than 80% sequence identity between any two species [37]. These empirically determined sequence identity thresholds were chosen to avoid overly-conserved regions that would introduce redundancy and cause overfitting, as well as overly-divergent regions that would unlikely constitute functional FC enhancers [38], [39]. Therefore, these identity cutoffs should ensure the representation of functional TFBSs in the training set that correspond to the regulatory function of interest, and thus provide sufficient information for training an accurate classifier [26], [40]. This approach is also consistent with the flexible information display or billboard model of transcriptional enhancers, as proposed by Arnosti and Kulkarni [41]. Using these parameters, we identified 24 orthologous FC enhancer sequences from 6 of the 14 orthologous species based on compliance with our sequence identity constraints, bringing the total size of the training set to 40 elements (Table S1).

**Figure 2:**
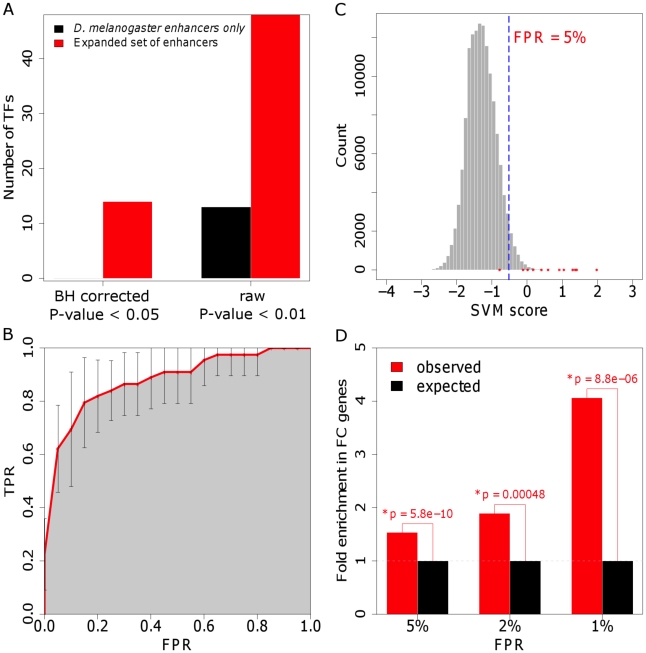
The enhancer classifier performs with high specificity and sensitivity. (A) Over-representation of TFBSs in the training set including only *D. melanogaster* enhancers and in the set extended using phylogenetic profiling, as compared with background sequence. P-values were adjusted for multiple testing using the method of Benjamini and Hochberg (BH) [120]. (B) Average ROC curve for the 10-fold cross-validation. Our method achieves an area under the ROC curve of 0.89 (shaded in gray). FPR: false-positive rate; TPR: true-positive rate. (C) Distribution of FC enhancer scores for the genome-wide scan. Scores assigned by the classifier for each evaluated sequence are shown in red. We used a FPR of 5% to define a cut-off for putative enhancers (dotted blue line; see Materials and Methods for details). (D) Fold-enrichment in 180 validated FC genes in the neighborhood of putative FC enhancers, as determined for different FPRs. Intergenic putative FC enhancers were associated with the closest gene, whereas intronic sequences were associated with their host gene. P-values were computed using the binomial test.

To confirm the validity of the phylogenetic profiling approach, we assessed the performance of different classifiers trained on subsets of 62 *Drosophila melanogaster* enhancers having activities in various mesodermal cell types that was retrieved from the REDfly database [32] and 72 of their orthologs (see Materials and Methods for details). The large size of this dataset, and the functional similarity of its members to the activities of the elements that are the focus of this study, allowed an accurate evaluation of the impact of phylogenetic profiling on the prediction performance across training sets of gradually increasing sizes (including 10, 15, 20, …, and 60 randomly chosen mesodermal enhancers). As expected, increasing the size of the training set improves the classification performance until approaching its maximum and thereby rising to an asymptote (Figure S2A). The improvement in the classification performance, measured by the area under the curve (AUC) of receiver operating characteristic (ROC), stabilizes for classifiers trained on approximately 40 elements, suggesting this to be the minimum necessary number of enhancers to train a reliable classifier. We also found that the addition of orthologous sequences to the training set significantly improves the performance of the 91% of the classifiers independently of the size of the training set (all P-values<0.05, Figure S2A) and reduces the error in the estimation of the true accuracy of all classifiers (Figure S2B). Furthermore, phylogenetic profiling improves the concordance between predicted outcomes, and thus, classifiers including enhancer orthologs systematically recognize a larger proportion of enhancers as compared with the classifiers trained only on *Drosophila melanogaster* enhancers (Figure S2C and S2D). Therefore, supplementary orthologs not only increase the prediction accuracy, but generate more stable classifiers, with more reproducible predictions. In addition, increasing the size of the training set by including presumably functional orthologous sequences that span different evolutionary distances increases our statistical power. For example, we identified over-represented binding sites of 14 TFs in the expanded set that included the orthologs and none in the original FC enhancer set (as compared with background sequence, correcting for multiple testing; Figure 2A). Among over-represented TFBSs are motifs for FoxO1, Ets and the MyoD family of TFs, which are known to play a role in muscle differentiation [42]. Overall, these results are consistent with what would be expected for an increase in the size of the training set [43]–[45], and support the use of phylogenetic profiling for expanding the training set.

**Figure 3:**
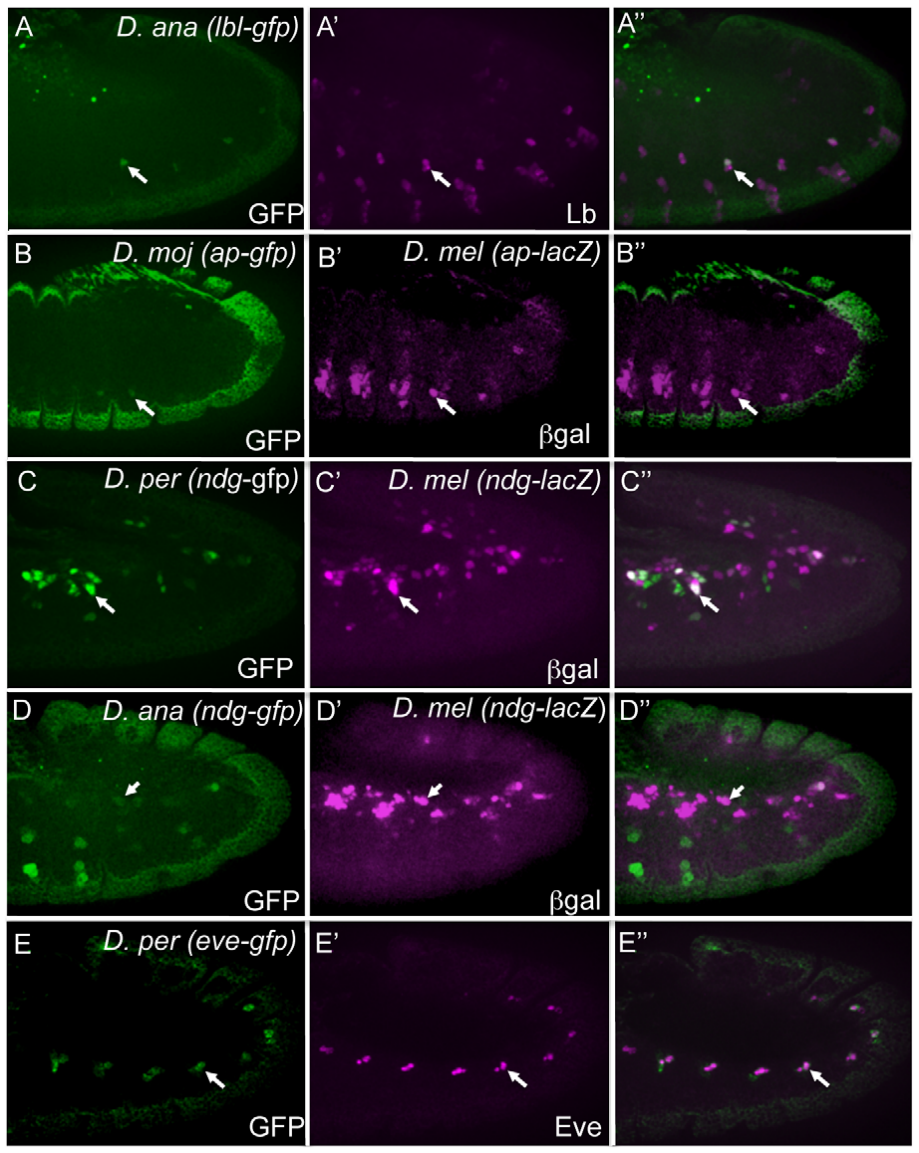
Orthologous sequences are functional enhancers. GFP (green) expression in transgenic stage 11 *D. melanogaster* embryos containing the indicated GFP reporter constructs driven by the *D. ananassae lbl* (A), *D. mojavensis ap* (B), *D. persimilis Ndg* (C), *D. ananassae Ndg* (D), and *D. persimilis eve* (E) enhancers. Co-expression of GFP driven by the *D. ananassae lbl* enhancer with endogenous Lbl protein (magenta, A′) and *D. persimilis eve* enhancer with endogenous Eve protein (magenta, E′). β-Gal driven by the *D. melanogaster* versions of the *Ndg* (C′, D′) and *ap* (B′) enhancer co-expresses in some but not all mesodermal cells with GFP driven by the orthologous sequences.

To verify that the orthologous sequences function as FC enhancers, we randomly chose 5 examples to test for transcriptional activity in *D. melanogaster* embryos using transgenic reporter assays. Each enhancer construct was introduced into the same *attP* site in the *D. melanogaster* genome using a custom vector containing a green fluorescent protein (GFP) reporter and an *attB* site allowing phiC31 integrase-mediated integration ([46], [47] and B. W. Busser, L. Shokri, S. A. Jaeger, S. S. Gisselbrecht, A. Singhania, M. F. Berger, B. Zhou, M. L. Bulyk and A. M. Michelson, unpublished data). All of the tested sequences drove similar, although not always identical, expression patterns as their orthologous *D. melanogaster* enhancers (Figure 3).

Of note, in several cases, we observed differences in the organization of TFBSs within *D. melanogaster* FC enhancers and their orthologs, which did not appear to affect their transcriptional activities. For example, the *D. melanogaster lbl* FC enhancer contains multiple binding sites for each of Pnt, Twi and Tin [5], whereas there are several Pnt, only one Twi and no Tin binding sites in the candidate *D. ananassae lbl* enhancer (Figure S3A). Nevertheless, both the *D. melanogaster* and *D. ananassae* enhancers direct reporter expression in the same two adult muscle precursors and single embryonic muscle FC in which endogenous *lbl* is expressed (Figure 3A). In this case, the cellular specificity achieved by the orthologous enhancer might be accounted for by the perfect conservation of a single binding site that is preferred by the Slouch (Slou) homeodomain TF, which we have recently shown to be critical in repressing activity of the *D. melanogaster lbl* enhancer in two Slou-expressing FCs (B. W. Busser, L. Shokri, S. A. Jaeger, S. S. Gisselbrecht, A. Singhania, M. F. Berger, B. Zhou, M. L. Bulyk and A. M. Michelson, unpublished data).

In other examples, the expression patterns driven by orthologous enhancers were similar but did not precisely replicate those of their *D. melanogaster* counterparts. For example, the *ap* muscle enhancer is active in a subset of endogenous *ap*-expressing muscles and was previously shown to depend on the input of Hox TFs [35]. Interestingly, only 3 out of the 5 known functional Hox binding sites are conserved between the *D. melanogaster* and the *D. mojavensis* orthologous sequences (Figure S3B). To compare activities of the orthologous enhancers, we generated a *D. melanogaster* transgenic line containing a *D. mojavensis ap-GFP* reporter construct and crossed it to a *D. melanogaster ap-lacZ* reporter strain. This experiment revealed that the candidate *D. mojavensis ap* enhancer is indeed active in muscle FCs, but only in a subset of the cells that express the reporter driven by the *D. melanogaster* enhancer (Figure 3B).

We also observed interesting patterns of TFBS reshuffling between the orthologs of some FC enhancers. For example, a 643 bp sequence in the first intron of the *D. melanogaster Nidogen* (*Ndg*) gene activates reporter expression in a subset of muscle FCs, pericardial and cardial cells of the heart, and cells of the central nervous system (Figure 3C, 3D and data not shown), and was originally identified based on the presence of binding sites for Pnt, Twi and Tin [5]. GFP reporter constructs of *Ndg* enhancer candidates from *D. persimilis* and *D. ananassae* were tested in transgenic *D. melanogaster* embryos. To compare the activities of the ortholgous enhancers, we crossed *D. persimilis Ndg-GFP* or *D. ananassae Ndg*-*GFP* reporter constructs to a *D. melanogaster Ndg*-*lacZ* reporter strain (Figure 3C and 3D). The orthologous enhancers co-activate their respective reporters in *D. melanogaster Ndg*-expressing FCs, albeit a minority, with extensive additional activity evident in other mesodermal cells. The finding of distinct expression patterns for all tested *Ndg* enhancer sequences is noteworthy as there is significant conservation of Pnt, Twi and Tin binding sites between *D. melanogaster* and *D. ananassae* but not *D. persimilis* versions of the *Ndg* enhancer (Figure S3C). This finding suggests that different ordering and spacing of TF binding sequences (both conserved and non-conserved) can be employed by an enhancer to activate gene expression in FCs and other mesodermal cells [41], although precise cellular specificity is dependent on a fixed arrangement of binding sites. We note, however, that such inferences are based entirely on sequence comparisons, and that a more detailed understanding of the significance of the apparent evolutionary shuffling of TFBSs would require extensive in vivo functional testing.

Finally, we observed variable ordering and distances between individual TFBSs among the orthologs of FC enhancers, as exemplified by *even skipped* (*eve*). This gene is expressed in two pericardial cells of the heart and a single dorsal somatic muscle FC [48]. Eve expression is positively regulated by the Wingless (Wg), Decapentapalegic (Dpp) and receptor tyrosine kinase (RTK)/Ras signaling pathways, and the gene is active in domains of the mesoderm in which Twi and Tin are critical [28], [49]. An enhancer that integrates these convergent inputs was isolated and shown to contain clusters of binding sites for T cell factor (Tcf), Mothers against dpp (Mad), and Pointed (Pnt), TFs acting downstream of Wg, Dpp and RTK/Ras signals, respectively, as well as binding sites for Twi and Tin [28]. Here we show that the orthologous *D. persimilis* sequence is expressed in an identical pattern (Figure 3E). Interestingly, the *D. persimilis eve* muscle and heart enhancer contains clusters of Tcf, Mad, Pnt, Twi and Tin binding sites, but the precise positions of these sites are generally not well conserved (Figure S3D). The orthologous *D. virilis eve* enhancer has a similar structure in which all 5 of these TF binding site classes are present [29].

In total, 5 out of 5 tested orthologous sequences drove expression in a pattern that is similar (*eve* and *lbl*), though often not identical (*Ndg*), to the *D. melanogaster* enhancer. The imprecise activities of some of the orthologous enhancers may reflect the partial level of sequence identity that could affect as yet unidentified binding sites, may result from the extensive shuffling of known binding sites for co-regulatory TFs, or might simply be a reflection of differential gene expression in the orthologous flies [26], [27], [40], [50]. Importantly, the general preservation of enhancer activity in the absence of extensive sequence conservation—a point which is further confirmed by the apparent shuffling of binding sites for known co-regulatory TF binding sites—suggests that these elements share other common sequence features. Thus, increasing the training set with orthologous sequences should minimize potential over-fitting caused by training on an otherwise small set of validated enhancers.

### Machine Learning of the FC Enhancer Code Results in an Accurate FC Enhancer Classifier

The FC training set consisted of 16 *D. melanogaster* FC enhancers plus 24 orthologous sequences. However, as previously noted, the activity of these enhancers is not restricted to FCs, with only 2 out of 16 tested enhancers displaying such localized activity (Table S1). Therefore, any computational model for FC enhancer classification will likely predict enhancers having broad mesodermal expression patterns that include but are not restricted to FCs. As a control set, we randomly sampled 1000 non-coding *D. melanogaster* sequences with length, GC- and repeat-content distributions similar to those of the FC training set. To discriminate between FC enhancers and other non-coding sequences, we modified a machine learning approach that was previously developed for the prediction of mammalian heart enhancers, with many of those results validated *in vivo* [7]. This method captures sequence patterns specific to a set of similarly acting non-coding sequences, relying on known TFBSs, as well as *de novo* motif discovery, to account for unascertained TF binding specificities. Known TFBSs were obtained from the literature and available databases (see Materials and Methods). *De novo* motif discovery was performed using PRIORITY [51], a Gibbs sampling approach that searches for over-represented motifs in a set of sequences.

With the aim of discovering TFs with critical roles in FC co-regulation, we assumed no prior knowledge of active TFs. Each sequence in the training and control set was represented by the number of occurrences per base pair of each of the 945 considered motifs. A linear Support Vector Machine (SVM) was trained to distinguish between FC enhancers and control sequences based on TFBS occurrences. The ability of the classifier to accurately predict regulatory activity was assessed by a 10-fold cross-validation procedure. The performance of the classifier was evaluated using the AUC, a value ranging from 0.5 (random classification) to 1.0 (perfect classification). The obtained AUC value of 0.89 indicates reliable detection of FC enhancers by the developed classifier (see Materials and Methods and Figure 2B).

We next applied the classifier for de novo discovery of FC enhancers in the D. melanogaster genome. We used a sliding window approach to score ∼140,000 overlapping non-coding 1000 base pair-long sequences spanning the complete genome. Keeping a low false-positive prediction rate (FPR) of 5%, approximately 5,500 sequences were annotated as putative FC enhancers (Table S3). Similar to what we observed for the training set, the individual conservation profile of the *D. melanogaster* putative FC enhancers generally reflects the phylogenetic distances of the species involved in the analysis, with most orthologs in the 50–80% sequence identity range in *D. yakuba*, *D. erecta*, *D. ananassae*, *D. pseudoobscura* and *D. persimillis*. However, putative FC enhancer sequences tend to be more deeply conserved than background genomic sequence (P-value<0.05, computed using the Binomial test, corrected for multiple testing using Bonferroni's method), and thus, probably functional (see Text S1). Although it was not surprising that the scores of the FC enhancers in the training set were positively-skewed (Figure 2C), it was reassuring to find that putative FC enhancers are strongly associated with genes that are expressed in FCs. For example, we found that at a FPR of 5%, 222 enhancer predictions are associated with 77 genes expressed in FCs, a number that is 1.5-fold higher than would be expected by chance (P-value = 5.8×10^−10^; Figure 2D). The latter result suggests that the sequence features learned by the classifier have specificity for FC enhancer function.

### Functional Assessment of Enhancers Predicted by the Classifier

**Figure 4:**
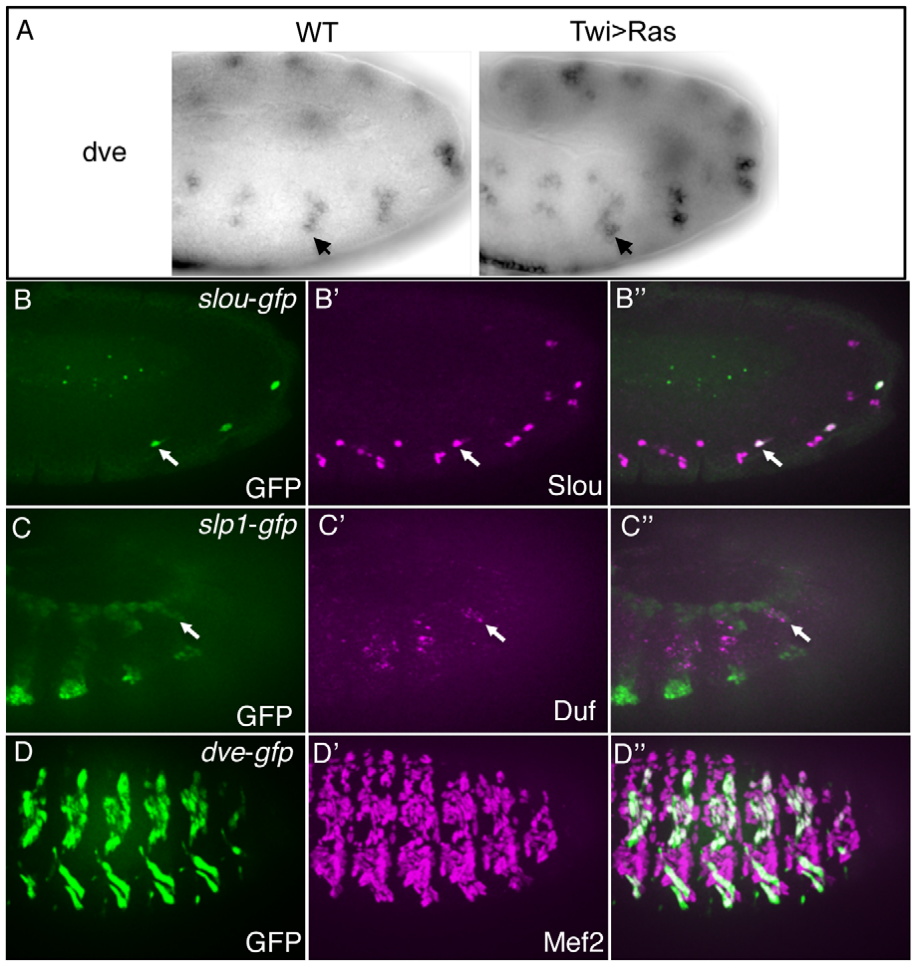
Candidate enhancers predicted by the classifier are active in FCs. *In situ* hybridization of *dve* in wild-type (WT) embryos and embryos over-expressing Ras (Twi>Ras) in the mesoderm (A). Note the increased activity of *dve* in Twi>Ras embryos, indicative of a FC gene [22]. GFP driven by the classifier-predicted enhancers associated with the upstream sequences of *slou* (arrows in B) and *slp1* (arrows in C). Slou protein (magenta) co-expresses with GFP (green) in *slou-GFP* embryos (B). Duf (magenta), which marks all FCs, co-expresses with *slp1*-*GFP* (green) (C). GFP (D) driven by the classifier-predicted intronic sequence associated with the *dve* gene co-expresses with Mef2 (D′) in myotubes at stage 15 in *dve-GFP* embryos.

To test the functions of the classifier-predicted enhancers, we first asked if the presence of a high-scoring putative FC enhancer could be used to predict expression in FCs [52]. To more readily associate an enhancer with its putative target gene, we examined the expression patterns of genes with a high-scoring intronic enhancer, which was assumed to control the gene in which it is located. In total, 5 genes out of 20 tested (25%) were actually expressed in FCs (Table S3). This is 8-times higher than would be predicted by chance (P<0.002), given that only 3% of *Drosophila* genes have been estimated to be expressed in FCs [22]. As an example, *defective proventriculus* (*dve*) was identified and validated as a novel FC gene using this approach (Figure 4A). In summary, since the presence of putative FC enhancers is strongly associated with FC gene expression, it is likely that a large fraction of FC enhancer predictions represent authentic FC regulatory sequences, including FC-specific enhancers and possibly silencers and insulators [2].

To directly assess the *in vivo* functions of these candidate enhancers, we used site-specific transgenic reporter assays to test 12 enhancer predictions associated with known FC genes. We assayed the activities of genomic regions with varying scores in the classifier ranking (Table S3). Whereas 9 out of the 12 candidates were found to have enhancer activity, 4 of these were functional in the mesoderm, with 2 directing reporter expression in muscle FCs (Figure 4). Forty-four percent (4/9) of enhancers driving expression in mesoderm represents a validation rate comparable with p300 based ChIP-Seq discovery of tissue-specific enhancers [53], while 2/9 FC enhancers in the set was below expected. These findings presumably reflect the limitations of the training set which, as previously noted, contain only 2 enhancers with specificity restricted only to FCs. Other factors contributing to this outcome are considered in the Discussion.

One informative example of a newly identified FC enhancer is that associated with *slou* (Figure 4B). This enhancer is found upstream of the gene in a region previously shown to recapitulate the complete FC expression of *slou* [54], but it is active in only a subset of all *slou*-expressing cells (in particular, those which correspond to the lateral oblique 1 (LO1) and ventral transverse 1 (VT1) muscles). This result suggests that additional regulatory elements must account for the complete expression pattern of this FC gene [55], unlike the situation for the majority of FC enhancers. The predicted enhancer associated with *slp1* is also located upstream of the gene and directs reporter activity both to FCs (Figure 4C) and to mesodermal and ectodermal stripes which are known to express *slp1* [56]. Of note, the intronic enhancer for *dve*, a gene which was tested for expression in FCs based on the presence of this predicted FC enhancer (Figure 4A), was not active during the FC stage of myogenesis but did direct reporter expression slightly later when myotubes develop (Figure 4D). It remains possible that the activity of this element occurs at the FC stage but is insufficiently strong to be detected by the present assay. Alternatively, a separate enhancer may be directing the early FC activity of *dve*, consistent with the additional candidate enhancers associated with this gene (Table S3). In this case, the classifier appears to be detecting features shared by early- and late-acting muscle enhancers without discriminating FC-specific elements, which is not surprising given that many TFs are expressed and active in the same cell types at different stages of development [20], [54]. Thus, while the classifier has some predictive value for FC enhancers, the regulatory network specifying these cells is sufficiently diverse and complex that the available training set is insufficient to provide a higher success rate for identifying new FC enhancers. To begin unraveling the complexities of this network, we need to define a more extensive collection of myogenic transcription factors and the DNA sequences to which they bind. To this end, we turned to an examination and validation of the novel sequence motifs detected by the classifier.

### Identification of Novel Sequence Motifs within FC Enhancers

**Figure 5:**
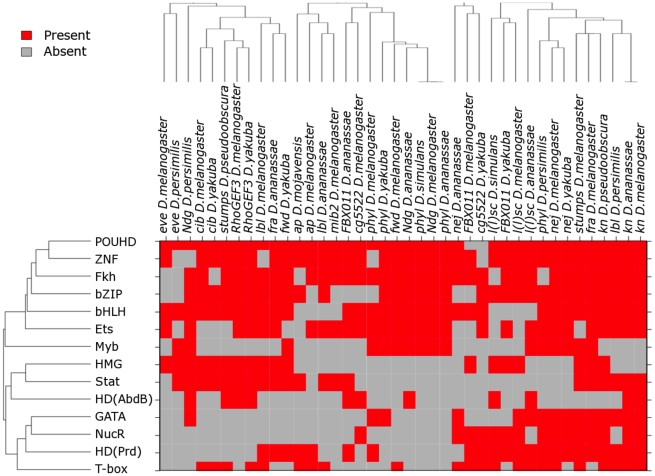
DNA binding domains of the TFs most relevant to FC enhancer classification. Only DNA binding domains for the fifty most relevant TFs have been included. TFs were ranked according to the SVM weights of their respective motifs, which represent their discriminating power. Only the highest scoring motif for each TF was considered (median ranks computed across 10 random partitions of the training data varied between 12 and 117). *De novo* motifs were explicitly excluded from this analysis. TF domains and sequences have been clustered using average linkage and Euclidean distance. The dendogram on top of the heatmap represents the relationships among the sequences in the training data, built on the presence/absence of TFBSs recognized by a specific class of TF DNA binding domain. The dendogram on the left of the heatmap shows the relationships among the different TF DNA binding domains.

To begin constructing a more comprehensive myogenic network, we examined the sequence features associated with the computational classification of FC enhancers. These features included position weight matrices of known TF binding specificities found within the TRANSFAC database, as well as motifs not represented in this database that can be identified by the PRIORITY algorithm [51]. In the case of linear SVMs, features irrelevant to the classification receive zero weight, whereas those associated with the signal and control set receive positive and negative weights, respectively (see Materials and Methods). Since a finite number of TFs is expected to regulate FC gene expression, only some of all possible motifs will be relevant to the classification. Indeed, out of the original 945 features, 200 contributed to approximately 50% of the weights in the decision function of the classifier, suggesting their importance in the prediction of FC enhancers. Sixty-percent of these 200 motifs were associated with positive weights and correspond to almost 60 distinct TFs (Figure S4 and Table S4). Most of these TFs belong to only a few families having similar binding profiles, which we are unable to individualize (Figure 5).

This diverse compilation of motifs suggests that the motif signature of FC enhancers is complex. However, this interpretation should be considered with caution, since training on a set of enhancers with diverse expression (Table S1) is likely to lead to the identification of multiple enhancer signatures. In any case, we were encouraged by the fact that known myogenic regulatory motifs, including Ets, Mef2 and MyoD (due to similarities in binding preferences, E-boxes may represent motifs for Twi, MyoD or other TFs having basic-helix-loop-helix DNA binding domains), are among those with the highest discriminatory power. Other identified motifs, including those for Stat [57] and homeodomain proteins [35], [58], [59], appear to play critical roles in myogenesis. In addition to known TFBSs, *de novo* motifs make a key contribution to the classification and presumably account for binding sites of TFs missing from TFBS libraries, or constitute more accurate representations for the binding specificities of incompletely characterized TFs (Figure S5). In particular, we found that the most relevant *de novo* motif represents the binding specificity of Tin [60], consistent with the well-established mesodermal regulatory functions of this TF [61].

### Predicted Motifs Regulate Enhancer Function in FCs

**Figure 6:**
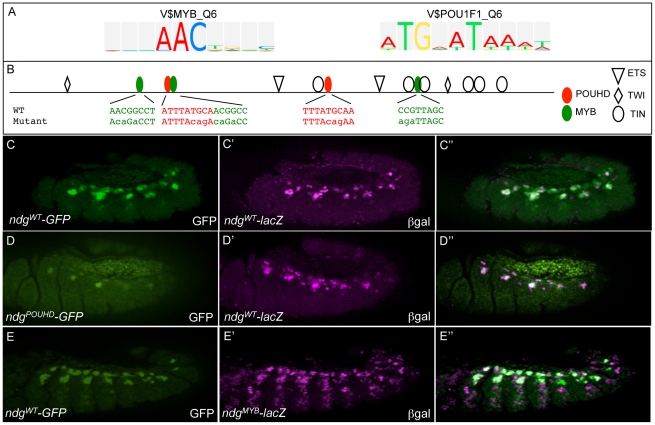
The wild-type activities of FC enhancers require input from classifier-defined Myb and POUHD TF binding motifs. (A) TRANSFAC position weight matrices for Myb (V$MYB_Q6) and POUHD (V$POU1F1_Q6) enriched motifs identified by the classifier. (B) Binding site sequences in the *Ndg* enhancer for Myb and POUHD and versions in which those sites are selectively mutated. Motifs were defined by searching for matches to the vertebrate homologues in the UniPROBE database [99]. The identification of these binding sites and the designs of the mutant versions are described in Table S4. (C) GFP (green) and β-Gal (magenta) are co-expressed when driven by the wild-type (WT) *Ndg* enhancer (*Ndg^WT^-GFP* and *Ndg^WT^-lacZ*, respectively). (D) GFP (green) expression driven by a version of the *Ndg* enhancer in which POUHD sites are selectively inactivated (*Ndg^POUHD^-GFP*) is significantly reduced compared to β-Gal (magenta) driven by *Ndg^WT^-lacZ*. (E) β-Gal driven by a version of the *Ndg* enhancer in which Myb binding sites are selectively inactivated (*Ndg^Myb^-lacZ*) is de-repressed into additional somatic mesodermal cells compared to GFP driven by a WT version of the *Ndg* enhancer (*Ndg^WT^-GFP*).

To determine if the newly identified motifs are functionally relevant to FC gene expression, we employed site-directed mutagenesis of such putative binding site sequences in otherwise wild-type FC enhancers. We initially concentrated on the potential role of Ets, Myb, POU homeodomain (POUHD) and Fkh binding motifs (see Figure 5, Figure 6A, and Figure S7B). Each of these motifs is over-represented in both individual FC enhancers and their orthologous sequences when compared to controls (Figure S6). Sequence matches to Myb and POUHD motifs in the wild-type *Ndg* enhancer and a version in which the sites are mutated are shown in Figure 6B (also see Table S4). To compare activities of the different constructs, we crossed *Drosophila* strains containing wild-type or mutant enhancer transgenes driving different reporters (either *GFP* or *lacZ*) to each other.

Mutagenesis of all motifs affected activity of the reporter as compared to wild-type versions of the enhancer (Figure 6C). For example, elimination of POUHD binding sites (Figure 6D) from an otherwise wild-type version of the *Ndg* enhancer reduced or eliminated enhancer activity in subsets of cells which express wild-type *Ndg*-*lacZ* (Figure 6D), whereas mutagenesis of Myb motifs caused an extensive de-repression of the reporter into additional somatic mesodermal cells (compare Figure 6E and 6E′). In addition, we found that the activity of Ets binding sites is critical for the full activity of the *Ndg* enhancer (Figure S7A), as had previously been demonstrated for another FC regulatory element [28]. Finally, mutagenesis of the Fkh binding sites in the *apterous* (*ap*) FC enhancer lead to a complete loss of reporter expression in those FCs in which the wild-type enhancer is active (compare Figure S7C and S7D). Collectively, the present experiments validating the functions of specific TFBSs in known FC enhancers document the critical role played by classifier-defined motifs in regulating specific gene expression patterns.

### Identifying a Novel Myogenic Transcription Factor from Motifs Over-Represented in FC Enhancers

The preceding analyses indicate that the regulatory motifs learned by the classifier are critical for the normal functions of FC enhancers. Next, we used classifier results not only to discover a new *cis*-acting motif but also to identity the corresponding TF that binds to this sequence and to functionally characterize it as a previously unrecognized myogenic regulator.

**Figure 7:**
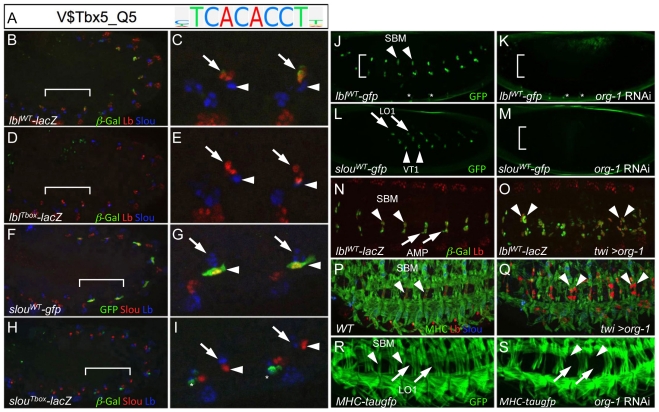
The T-box TF *org-1* is a regulator of the *lbl* and *slou* FC enhancers. (A) TRANSFAC position weight matrix for Tbx5 (V$Tbx5_Q5), a vertebrate homolog of the *Drosophila* T-box TF *org-1* and a top-scoring feature derived by the FC enhancer classifier. (B, C) In stage 11 embryos containing the *lbl* ^WT^-*lacZ* transgene, β-Gal (green) co-expresses with endogenous Lb (red) in three cells (arrow) but is absent from Slou-expressing FCs (blue, arrowhead). (D, E) Mutagenesis of T-box motifs in the *lbl* FC enhancer (*lbl* ^Tbox^-*lacZ*) results in an attenuation of β-Gal (green) reporter activity in the three Lbl-expressing cells (red, arrow). Wild-type and mutant T-box binding sites in the *lbl* FC enhancer are described in Table S4. (F, G) GFP (green) co-expresses with endogenous Slou (red) in two cells (arrowhead) but not in the three Lbl- expressing cells (blue, arrow) in stage 11 embryos containig the *slou* ^WT^-*gfp* transgene. (H, I) Mutagenesis of T-box motifs in the *slou* FC enhancer (*slou* ^Tbox^-*lacZ*) results in a marked attenuation of β-Gal (green, arrow) expression in two Slou-expressing cells (red, arrowhead). The asterisks denote de-repression of the *lacZ* reporter in cells of unknown identity. Wild-type and mutant T-box binding sites in the *slou* FC enhancer are described in Table S4. (J) GFP (green) fluorescence expression in living stage 14 *lbl-GFP* embryos is visible in the SBM (arrowhead), in two adult muscle precursors and in several cells of the central nervous system (asterisks) injected with control *lacZ* dsRNA. (K) Loss of GFP fluorescence from cells corresponding to the wild-type positions of the SBM and two adult muscle precursors but not in cells of the central nervous system (asterisks) in living stage 14 *lbl-GFP* embryos injected with *org-1* dsRNA. (L) GFP (green) fluorescence expression in living stage 14 *slou-GFP* embryos is visible in muscles LO1 (arrow) and VT1 (arrowhead) injected with control *lacZ* dsRNA. (M) Loss of GFP fluorescence from cells corresponding to the wild-type positions of LO1 and VT1 in living stage 14 *slou-GFP* embryos injected with *org-1* dsRNA. (N) Co-expression in the segment border muscle (SBM; arrowhead) of endogenous Lbl (red) and β-Gal in stage 14 *lbl^WT^-lacZ* embryos containing the *lbl^WT^-lacZ* transgene. (O) Panmesodermal expression of *org-1* (Twi>org-1) in stage 14 *lbl^WT^-lacZ* embryos induces ectopic activation of both endogenous Lb (red) and the β-Ggal reporter reporter (green). (P) Stage 16 wild-type (WT) embryo stained with antibodies directed against myosin heavy chain (MHC; green), Lb (red) and Slou (blue) showing expression of Lb in the single SBM (arrowhead) in each hemisegment. (Q) Panmesodermal expression of *org-1* (Twi>org-1) induces duplication of the SBM in some but not all hemisegments (arrowheads). (R) GFP (green) fluorescence expression in living stage 16 MHC-*tauGFP* embryos is visible in the SBM (arrowhead) and muscle LO1 (arrow) injected with control *lacZ* dsRNA. (S) Loss of GFP fluorescence from cells corresponding to the wild-type positions of the SBM muscle LO1 in living stage 16 *MHC-tauGFP* embryos injected with *org-1* dsRNA.

One of the top-scoring classifying features of the enhancer training set was a motif that binds to T-box TFs (Figure 5, Figure 7A, Figure S4, and Figure S6). This finding could either reflect the existence of a novel myogenic regulator or, since the training set of FC enhancers also contain many elements with heart activity (Table S1), it could simply indicate the functions of known cardiogenic T-box TFs [62]–[64]. To distinguish between these possibilities, we first defined the expression pattern of every *Drosophila* T-box TF (Table S5), which confirmed that *Dorsocross 3* (*Doc3*) and *optomotor-blind-related-gene-1* (*org-1*) are the only T-box TFs expressed in muscle FCs [22], [62]. In particular, *org-1* is co-expressed with Slou in the FCs corresponding to muscles LO1 and VT1, and with Lbl in the FC for the segment border muscle (SBM) (Figure S8).

The previous co-expression studies raise the possibility that *org-1* may directly regulate *slou* and *lbl*. To test this hypothesis, we identified potential T-box binding sites in the *lbl* and *slou* FC enhancers (Table S4). The previously described *lbl* muscle enhancer is active in the SBM and in two adult muscle precursor cells [5], while the *slou* FC enhancer identified in the present work is active in the two FCs which become muscles LO1 and VT1 (Figure 4B). Of note, the *slou* FC enhancer was predicted by the classifier due to the presence of a combination of motifs, including those that bind to T-box TFs. Targeted mutagenesis of the T-box sites in otherwise wild-type *lbl* (Figure 7D and 7E) and *slou* (Figure 7H and 7I) enhancers revealed that these sites are essential for full enhancer activity (compare to the wild-type versions in Figure 7B, 7C and 7F, 7G respectively). These results suggest that Org-1 is a direct activator of *slou* and *lbl* expression in these three FCs. If this is the case, then *org-1* loss- and gain-of-function should lead to decreased and increased expression, respectively, of the putative target genes [65]. In agreement with this expectation, RNAi-mediated knockdown of *org-1* causes loss of *lbl*-*GFP* (Figure 7K) and *slou-GFP* (Figure 7M) activity, whereas panmesodermal overexpression of *org-1* is associated with ectopic activation of both the endogenous *lbl* gene and the *lbl* enhancer-driven reporter (Figure 7O), as well as duplication of the SBM in late-stage embryos (Figure 7Q). These results suggest that Org-1 is a direct regulator of *lbl* and that it also contributes to the development of the *lbl*-expressing muscle. Consistent with the latter prediction, RNAi-mediated knockdown of *org-1* in embryos expressing tau-GFP under control of a myosin heavy chain enhancer revealed a loss of both the SBM and muscle LO1 (Figure 7S). In summary, our computational enhancer classification not only led to the discovery of a T-box regulatory motif, but also facilitated the identification of *org-1* as encoding a TF critical for FC enhancer activity and for determining muscle FC identity.

### TFBS Composition of FC Enhancers

Having identified and experimentally validated the functions of 4 novel TFBSs that we found to be over-represented in FC enhancers—POUHD, Myb, Fkh and T-box—we were next interested in determining the distribution of all known regulatory motifs in enhancers of this class. We reasoned that such a survey might reveal whether TF combinatorics contribute to FC enhancer specificity. Thus, we analyzed all 18 *D. melanogaster* FC enhancers (16 from the original training set plus 2 more enhancer predictions whose activities were validated in the current study) for the presence of a total of 11 types of TFBSs that are known to contribute to FC activity. For this purpose, we added 7 motifs from prior studies of FC enhancers to the 4 new motifs discovered here.

We had previously constructed and validated a regulatory model of FC enhancer activity which reflected the coordinated input of Tcf, Mad, Pnt, Twi and Tin [28], [29]. Subsequently, combining the clustering of FC genes based on genetic perturbation responses with a systematic *in silico* evaluation of candidate transcriptional regulatory models, we demonstrated that Pnt, Twi and Tin alone target a subset of highly Ras-responsive FC genes [5]. In addition to these 5 motifs, we included 2 other previously characterized myogenic regulatory sequences that are bound by Mef2 [66] and homeodomain (HD) TFs ([35], [67] and B. W. Busser, L. Shokri, S. A. Jaeger, S. S. Gisselbrecht, A. Singhania, M. F. Berger, B. Zhou, M. L. Bulyk and A. M. Michelson, unpublished data).

**Figure 8:**
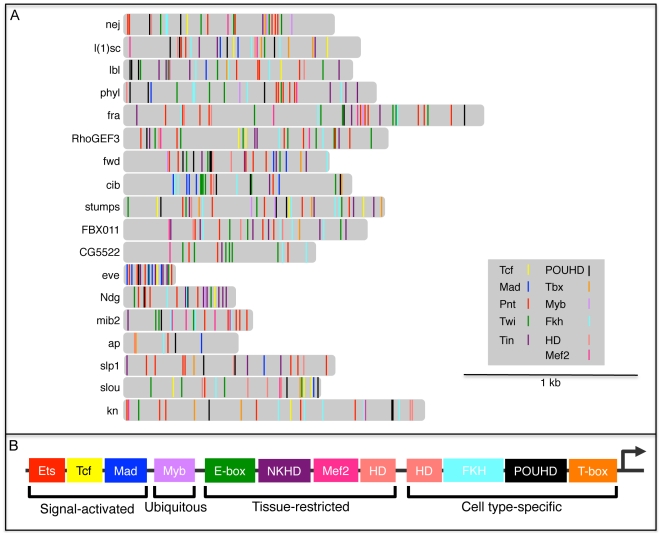
TFBS combinatorics within FC enhancers. (A) Distribution of Tcf, Mad, Pnt, Twi, Tin, POUHD, Tbx, Myb, Fkh, HD and Mef2 TFBSs in FC enhancers. Binding sites for Tcf, Mad, Pnt, Twi and Tin were previously published [5]. Motif matches for motifs most relevant to the classification for a given DNA binding domain class: POUHD (V$OCT_01, V$POU1F1_Q6, V$OCT4_02), Tbx (V$TBX5_01, I$BYN_Q6), Myb (V$MYB_Q6), Fkh (V$FOXO3_01, V$FOXO1_Q5, V$FREAC2_01), HD (I$ABDA_Q6, V$CDX5_Q5, V$IFP_03, V$PAX4_02), and Mef2 (V$AMEF2_Q6, V$HMEF2_Q6). These sites were mapped using MAST under default parameters [118]. (B) A generic FC enhancer receives differential input from signal-activated, ubiquitous, tissue-restricted and cell type-specific TFs. HD binding motifs are represented as both tissue-restricted and cell type-specific classes since these motifs receive input from both Hox TFs, which are widely expressed in the mesoderm [35], [59], [67], and muscle identity HD TFs—such as Slou, Msh and Ap—which are cell type-specific [54], [68], [69]. For this diagram, HD binding sites were not subdivided into the distinct binding profiles that have been identified for each individual HD TF ([83], [126] and B. W. Busser, L. Shokri, S. A. Jaeger, S. S. Gisselbrecht, A. Singhania, M. F. Berger, B. Zhou, M. L. Bulyk and A. M. Michelson, unpublished data).

Using position weight matrices (PWMs) for 3 signal-activated TFs (Tcf, Mad and Pnt), the ubiquitously expressed Myb, 4 tissue-restricted TFs (Twi, Tin, Mef2 and HD, where HD in this case represents Hox factors that are widely expressed throughout the somatic mesoderm [59]), plus 4 cell type-specific TF classes (POUHD, Fkh, T-box and HD, where in this case HD refers to muscle identity TFs such as Slou, Ap, and Muscle Segment Homeobox that are expressed in various subsets of FCs [54], [68], [69]), we scanned and scored all 18 FC enhancers for at least one occurrence of each of these 11 binding site motifs (Figure 8, Figure S9 and Table S6). Interestingly, this analysis revealed that each FC enhancer has a unique combination of predicted binding sites for all 11 of these TF classes. On the other hand, the FC enhancers exhibited various overlapping TFBS combinations when subsets of the 11 motifs were considered (Figure S9 and Table S6). Of note, the only motif that is present in all 18 FC enhancers binds the MAPK-activated TF Pnt, a result that is consistent with prior evidence demonstrating that the receptor tyrosine kinase/Ras pathway is the major inductive signal for establishing all FC fates [49], [70].

A number of caveats must be considered in interpreting the above analysis of motif distributions within FC enhancers. First, except for the small number of cases where individual motifs have been functionally validated [28], [29], [35], [67], each motif occurrence corresponds to a computational prediction without a verified assignable function. Second, the probability of finding a motif match is increased in longer genomic sequences, whereas the minimally active region has not been determined for most enhancers in this set. One notable exception is the enhancer directing FC expression of *eve*, where a minimally active regulatory element has been defined. In the case of *eve*, an approximately 300 bp sequence contains multiple instances of 6 different TFBSs (Figure 8 and Table S6), all 6 of which have been functionally validated as contributing to FC activity [28], [29]. Third, PWMs, which are critically dependent on particular thresholds to limit false positives and negatives, were used to identify motifs within each enhancer. Fourth, the various PWMs have different relative information contents (Table S6), a parameter which affects the likelihood that a match will be found in any given sequence. Fifth, since many of these enhancers are active in cell types other than FCs, not all motifs that are present will necessarily contribute to FC activity. Notwithstanding these potential limitations, the present results suggest that the specificity of enhancer activities observed at the level of individual muscle FCs is reflected in the diversity of the TFBS compositions of these regulatory elements.

## Discussion

### Prediction of Enhancers

There are three main approaches for the prediction of tissue-specific regulatory elements that are based on high-throughput sequencing coupled with chromatin immunoprecipitation (ChIP-Seq), DNA sequence pattern analysis, or hybrid methods that combine both of these strategies. ChIP-Seq for p300 using mouse embryonic tissue has proven to be an accurate means for identifying enhancers and their associated activities, with *in vivo* validation rates varying from 62% to 88% [53], [71]. Computational analysis of whole-genome histone modification profiles using hidden Markov models [72], [73] and machine learning techniques [74] has also been highly successful at linking chromatin signatures with regulatory elements. Finally, computational models that identify tissue-specific enhancers relying on sequence motifs and linear regression and support vector machines have been similarly effective, with *in vivo* validation rates of *de novo* predictions ranging from 62% for heart enhancers [75] to 91% for brain enhancers [Taher et al., unpublished data]. Although experimental techniques are often preferred for identifying enhancers on a genome-wide scale, ChIP-Seq has several limitations. For example, ChIP-Seq experiments are typically carried out in only one species and for individual cell types, and are currently not sufficiently precise for low-quality genome sequences. Thus, *de novo* prediction of regulatory elements based on ChIP-Seq data critically depends on the availability of relevant data for the species, cell type and genomic regions of interest. Currently, computational analysis of DNA sequence patterns shared by a set of regulatory elements with the same or similar biological activity remains a highly effective method for the *de novo* discovery of tissue-specific enhancers, and the simultaneous elucidation of cell type-specific regulatory codes. The method presented in this study further extends the usefulness of computational sequence analysis by exploring phylogenetic information that can be used to improve the classification accuracy, a strategy that promises to be advantageous in the large number of cases where comparative genomics data are available.

Computational approaches for predicting *cis*-regulatory modules are commonly based on machine learning of arrangements of TFBSs in enhancers that have common functions [7], [10], [11], [13], [15], [76]. These methods rely heavily on a training set of related enhancers to detect over-represented TFBS combinations. Unfortunately, in the vast majority of cases—including the present study of *Drosophila* muscle FC enhancers—the size of the training set is limited by the lack of experimentally validated tissue- and cell type-specific enhancers, which results in overfitting of computational models and poor accuracy of predictions. To overcome this problem, and to provide a generalizable approach for increasing the size of the training set, we developed a phylogenetic profiling strategy based on a search for diverged orthologous counterparts of available enhancers from distantly related species. Twenty-four *Drosophila* orthologs were identified using this approach, which more than doubled the size of the training set. We assessed the ability to accurately distinguish FC enhancers in a cross-validation framework using the extended training set, and determined that the classifier accuracy is 89% as assessed by the AUC approach. We then applied this classifier to scan the entire genome of *D. melanogaster* for novel FC enhancers, retrieving 5,500 high-scoring predictions at a FPR of 5%. These predictions were significantly associated with genes expressed in FCs, demonstrating that the model was able to capture essential features of FC gene co-regulation. A similar machine learning approach could be applied to a diverse array of datasets, including experimentally-verified regulatory elements from co-expressed targets at either a germ layer, organ, tissue or cellular level from invertebrate and vertebrate databases [32], [33], [77], [78]. Alternatively, a similar approach could be coupled to a training set of predicted regulatory elements derived from genome-wide analyses of chromatin marks or DNAse hypersensitive sites in active enhancers associated with a co-expressed gene set [79], [80].

### Increasing a Small Training Set with Orthologous Sequences

Evolutionary constraint of functional sequences is routinely employed as an effective filter to improve the prediction of regulatory elements [13]. Furthermore, cross-species comparisons have been successfully exploited to obtain evidence for functional TFBSs. For example, Rouault *et al.* [76] used twelve *Drosophila* species to identify over-represented motifs in the regulatory elements of genes expressed in neural progenitor cells, with sequence orthologs used to enrich the training set and to give prominence to conserved motifs. However, our method extends this approach by including suitably diverged orthologous enhancers from other *Drosophila* species in the dataset used to train the classifier. Our purpose in designing this strategy was two-fold. First, we wanted to enrich for relevant sequence motifs in the training data, allowing for a level of variation that would improve the generalization of the model. Second, we wanted to provide a potentially wider variety of TFBS arrangements that characterize the architecture of authentic FC enhancers. In essence, the addition of orthologous sequences boosts the statistical power of the significance tests, revealing patterns of TFBSs that otherwise could have been neglected.

Of note, when 5 of these orthologous sequences were tested in transgenic reporter assays in *D. melanogaster*, the overall expression pattern generated was similar to the *D. melanogaster* counterpart despite extensive evolutionary shuffling of known TFBSs. Similar binding site reorganization has been documented for the enhancers that regulate both the segmentation and mesodermal patterns of *eve* expression [40]. Numerous other studies have shown that the order and spacing of TFBSs is critical for enhancer function [23]–[27], [50]. These results suggest that regulatory elements can direct similar expression patterns provided that the overall composition and order of collaborating TFs is maintained [40]. Our finding that enhancer function is preserved in the orthologous sequences examined here establishes the validity of the sequence conservation thresholds chosen for the present studies, and suggests that the incorporation of orthologous sequences to increase a training set without over-fitting the data will be a generally applicable approach.

### *In Vivo* Functions of Predicted Enhancers

To assess the accuracy of our method, we selected 12 predicted FC enhancers and tested their *in vivo* functions. Seventy-five percent of the putative enhancers were experimentally validated as having transcriptional activity, demonstrating the effectiveness of our approach to identify regulatory sequences. However, of the sequences showing regulatory functions, only 4 of 9 were active in the mesoderm—including 2 in FCs—and 3 of 9 had nervous system activity. These data suggest that our model has been able to reliably recognize general properties of tissue-specific enhancers without specifically distinguishing an overall muscle FC code, even though numerous individual FC-specific motifs were identified (see below). The former finding is similar to the results of Sinha and colleagues [13] who found that the majority of their classifier predictions were active enhancers, but only a minority were expressed in the predicted pattern. A number of confounding factors can explain this outcome.

First, most members of the enhancer training set are active in both FCs and other cell types, including additional mesodermal cells such as the cardiac and visceral mesoderm, as well as some cells of the nervous system. For example, the enhancer responsible for the FC activity of the *hunchback* gene is also active in the longitudinal visceral mesoderm, and enhancers directing the FC expression of the *vestigial*, *big brain* and *king-tubby* genes are also active in the peripheral nervous system (Table S3). These results suggest that the regulatory networks specifying the somatic and visceral mesoderm share common features, which is consistent with both the available genetic and genomic evidence for the diverse developmental functions of key mesodermal transcription factors [81], [82]. Second, different members of a given TF family bind to similar motifs but have distinct tissue-specific expression patterns and developmental activities. Thus, combinations of motifs involved in the specification of muscle FCs and the nervous system may overlap. For example, this situation occurs with E-box and NK-homeodomain motifs [5], [58], [76], [83], [84]. Third, some TFs are expressed and functional in the derivatives of more than one germ layer [54], [85]. Fourth, the sequence features characteristic of cell type-specific enhancers, such as those active in muscle FCs, are expected to be under-represented in available training sets owing to the diversity of combinatorial TF models required to specify such a heterogeneous cell type [18], [20]. Identification of many examples of a particular cell-specific signature is a major challenge since each of the approximately 30 FCs in each *Drosophila* hemisegement expresses a unique combination of cell-specific muscle identity TFs and downstream target genes [18], [19]. Thus, 30 distinct cell states exist, each governed by a different but partially overlapping set of regulatory TFs. In contrast to the difficulties involved in dissecting regulatory codes at single cell resolution, shared features that direct activity to the general level of tissues and organs have been more readily identified using a machine learning approach, as was found here for enhancers having mesodermal, although not necessarily FC, activity. This likely reflects the dominant role that some TFs play in the regulatory network specifying the identities of numerous tissues [86]–[91]. Fifth, since there appears to be a regulatory signature for enhancers [16], [92], it is likely that these aspects of enhancer structure will be more significantly over-represented than those features that specify individual FC activity patterns. Sixth, the use of phylogenetic profiling might have expanded the biological function of the training dataset by introducing additional enhancer functions acquired by the orthologs of the original *D. melanogaster* sequences during their evolution. While we have been able to show that the phylogenetic profiling approach improves the accuracy of the classifier, one drawback of its use might be that the final classifier recognizes a broader biological domain than the function of the original training set of sequences derived from the reference species. Finally, classifier predictions may represent *cis*-regulatory elements other than enhancers, for example, silencers and insulators [2], which would not be detected by our transgenic reporter assays.

In summary, a number of confounding factors influenced our ability to identify an enhancer signature that is specific for individual muscle FCs. However, despite these challenges, our successful identification of novel TF binding motifs responsible for the cell type-specific activity of FC enhancers encourages us that this is a tractable problem that can be solved by an iterative approach to the computational analysis of this and other complex developmental systems. Thus, future studies must focus on obtaining a larger training set of sequences in which enhancers are categorized based on their activities at single cell resolution, combined with the appropriate weighting of newly validated motifs that contribute to the expression pattern of interest. In this manner, each experimental round would improve the accuracy of the classifier.

### Sequence Motifs Associated with FC Enhancers Are Functional and Can Be Used to Identify Novel *Trans*-Acting Factors

The motifs ranked by our classifier as having the highest discriminatory power are part of a large regulatory network that is known to be critical for mesoderm specification and myogenesis. These motifs include binding sites for JAK/STAT [57], Ets [93], bHLH [94], [95], Wingless/Tcf [49], [96], [97], Mef2 [66], homeodomain ([19], [20] and B. W. Busser, L. Shokri, S. A. Jaeger, S. S. Gisselbrecht, A. Singhania, M. F. Berger, B. Zhou, M. L. Bulyk and A. M. Michelson, unpublished data) and forkhead (X. Zhu, S. M. Ahmad, A. Aboukhalil, B. W. Busser, Y. Kim, T. R. Tansey, A. Haimovich, N. Jeffries, M. L. Bulyk, and A. M. Michelson, unpublished data) proteins. Furthermore, we previously suggested that Ets is part of a transcriptional code regulating the C1 subset of FC genes [5], which we validated here using site-directed mutational analysis of the *Ndg* enhancer, a previously characterized regulatory element associated with a C1 FC gene.

To extend the components of the myogenic regulatory network beyond these known TFs and motifs, we examined the function of the classifier-defined sequence motifs recognized by POU homeodomain and Myb proteins, transcription factors having no previously known role in *Drosophila* myogenesis. Mutagenesis of POUHD motifs attenuated the activity of the *Ndg* enhancer in many mesodermal cells. However, a zygotic loss-of-function mutation in *acj6*, the only POUHD that we found to be expressed in the mesoderm, had no effect on *Ndg* gene expression (data not shown). Given the strong maternal contribution to this gene [98], we used RNAi to knock down both maternal and zygotic *acj6* transcripts, but this manipulation had no effect on *Ndg-GFP* reporter activity (data not shown). These findings leave unresolved the identity of the TF that binds to the motif in question. The future characterization of this TF, including exploring the possibility that it is not a POUHD protein, will require searching functional motifs against larger TF databases [99] or with STAMP [60], combined with analysis of the embryonic expression and function of any new candidates that emerge.

Inactivating mutations of the Myb binding sites in the *Ndg* enhancer led to extensive de-repression of the reporter in other mesodermal cells. Myb is a ubiquitously-expressed DNA binding protein which plays a critical role in controlling regulatory decisions during proliferation and differentiation of progenitor cells [100]. Identifying a putative role for Myb in myogenesis documents the power of this approach, since functional studies tend to focus on genes with restricted expression patterns. However, a definitive assessment requires examining the effect of loss-of-function mutations in *Myb*. In any event, as myogenesis in *Drosophila* occurs through a series of asymmetric and symmetric cell divisions [101], a role for Myb in regulating FC gene expression is entirely consistent with a transcriptional regulator acting at the interface between replication and transcription [102], [103]. Alternatively, Myb may cooperate with other TFs to activate cell or tissue-specific gene expression [104].

Interestingly, T-box motifs scored well in the classification, yet no role for T-box TFs has previously been described in *Drosophila* somatic muscle development, despite widespread functions of this TF class in mesoderm specification and myogenesis in vertebrates [105], [106], as well as cardiogenesis in *Drosophila* and vertebrates [62], [63], [107]. Here we show using both *cis* and *trans* tests of TF function, along with gene co-expression, that Org-1 is a muscle identity TF. In particular, the *cis* effects of Org-1 were documented in the FC enhancers associated with two known muscle identity TFs, Slou and Lbl, and *org-1* expression localizes to the SBM and VT1, muscles in which the *lb* genes and *slou*, respectively, are the only previously described determinants of muscle identity [54], [108]. *Slou* function is critical for the proper development of muscles LO1 and VT1 and is further required to repress the *lb* genes in these cells, suggesting a co-regulatory relationship between *slou* and *lb* [54]. It is likely that *org-1* acts upstream of *slou* and *lb* in this regulatory hierarchy since *org-1* expression precedes *slou* and *lb*, and the ectopic expression of *org-1* causes increased expression of *slou* and *lb* (Figure 7 and data not shown). In addition, the essential role of *org-1* in this regulatory network is revealed by the effects of *org-1* overexpression and RNAi knockdown on development of *lb*- and *slou*-expressing muscles. Interestingly, the mouse orthologs of *org-1* and *lb* genes, *Tbx1* and *Lbx1*, respectively, have been suggested to regulate myogenic differentiation in the limb [109]–[111]. Given the high degree of sequence similarity, and the close correspondence of expression patterns and functions in *Drosophila* and mouse, the collaborative roles of these two TFs in myogenesis appear to have been conserved through evolution.

### Motif Combinatorics in FC Enhancers

Computational prediction of regulatory elements requires a thorough understanding of the TFs and motifs that orchestrate gene co-expression patterns. In prior studies, we established that 5-way and 3-way “AND” combinations of 3 signal-activated (Tcf, Mad and Pnt) plus 2 tissue-restricted (Twi and Tin) TFs constitute distinct regulatory models for different FC enhancers [5], [28], [29]. The present study significantly extend these prior combinatorial codes for FC gene regulation by identifying four additional classes of TFBSs that are critical for accurate FC enhancer activity, namely POUHD, Myb, Fkh and T-box motifs. Moreover, these findings provided us with an opportunity to examine the complete spectrum of regulatory motif usage across a collection of regulatory elements that are active in different muscle FCs, which led to the identification of 18 unique combinations of 11 TFBSs for the entire set of 18 known FC enhancers. Thus, unlike other cases that have been studied, a single enhancer archetype does not appear to exist for this subpopulation of myoblasts [23]–[25], [50]. This finding likely reflects the fact that although these elements all display FC activity, with some overlap at the level of individual cells, no two FC gene expression patterns directed by this enhancer set are identical.

The marked heterogeneity of FC enhancer architecture uncovered here reflects not only distinct combinations of various TF classes (including signal-activated, ubiquitous and both tissue- and cell type-specific TFs), but also diversity at other biological levels, including the unique identities of the thirty muscle FCs and their differentiated derivatives in each abdominal hemisegment, and the different gene expression patterns exhibited by those particular cells. Thus, TFBS combinatorics provide a plausible molecular explanation for the functional complexity of enhancers having related but non-identical activites at the resolution of individual cells in the context of the developing embryo.

### Conclusions

We have investigated the transcriptional regulatory network specifying individual muscle FCs using an integrated genomics approach that includes identification of orthologous enhancers, *de novo* motif discovery, classification of enhancer sequence features, empirical testing of candidate enhancers, and *cis*-*trans* tests of target gene regulation. We also have established that a small set of training sequences can be expanded with orthologous sequences [76]. Moreover, motifs learned by the classifier were empirically found to be critical for the appropriate spatio-temporal activities of FC enhancers, and suggested new candidate TFs in the myogenic regulatory network. Using this approach, we identified one such candidate TF, Org-1, as a novel muscle identity TF, and further found that no two enhancers with related activities contain the same combination of TFBSs. The tools and strategy used here can be readily applied to other cell types to identify the motifs and *trans*-acting factors regulating a set of co-expressed genes. Finally, we anticipate that an iterative application of this approach, which could include training on datasets of different epigenetic marks associated with active enhancers [18], [80], [112], [113] or previous ChIP studies of known mesodermally-relevant TFs [114], will lead to further refinements in the determination of cell type-specific transcriptional codes.

## Materials and Methods

### Fly Stocks

*Drosophila* stocks containing the following transgenes and mutant alleles were used: UAS-*org-1* (gift of G. Pflugfelder, Univ. Wurzburg, Germany), attP40 and *nos*-phiC31intNLS [115] (gift of N. Perrimon, Harvard University, USA), *lbl*-*lacZ* and *Ndg*-*lacZ* [5], *ap-lacZ* (gift of J. Botas, Baylor College, USA) [35], *acj6^6^* (gift of J. Carlson, Yale University, USA) [116], and *twi*-*Gal4* [49].

### Analysis of Transgenic Reporter Constructs and Embryo Staining

Enhancer regions were either synthesized *in vitro* (Integrated DNA Technologies, Coralville, IA, USA) or PCR-amplified and sequence-verified and then subcloned into the reporter vector pWattB-GFP (B. W. Busser, L. Shokri, S. A. Jaeger, S. S. Gisselbrecht, A. Singhania, M. F. Berger, B. Zhou, M. L. Bulyk and A. M. Michelson, unpublished data) or pWattB-nlacZ. The pWattB-nlacZ vector was constructed by cloning the EcoRI-SpeI fragment from a version of pH-pelican [117] in which nuclear lacZ replaced cytoplasmic lacZ into the EcoRI-SpeI sites in the pWattB-GFP vector. All constructs were targeted to attP40 [47] with phiC31-mediated integration [46], and homozygous viable insertion lines were obtained. Whole-embryo immunohistochemistry, *in situ* hybridization and fluorescent *in situ* hybridization with tyramide signal amplification (Invitrogen, Carlsbad, CA, USA) followed standard protocols [28]. Embryo collections for *twi*-*Gal4* UAS-*org-1* were incubated at 25°C. For fluorescent staining, the following antibodies were used: mouse anti-Ladybird early (Lbe) (1∶2500, gift of K. Jagla; Lbe and Lbl are co-expressed in the same mesodermal cells), rabbit anti-Slou (1∶200, gift of M. Baylies), chicken anti-GFP (1∶2000, Abcam, Cambridge, MA), mouse anti-βgal (1∶500, Promega, Madison, WI), rabbit anti-Kirre (1∶200, gift of K. Fischbach), rabbit anti-MHC (1∶500, gift of D. Kiehart), and guinea pig anti-Eve (1∶200, gift of D. Kosman).

### RNA Interference Assay

Embryo RNAi was performed as previously described [22]. Using SnapDragon (http://www.flyrnai.org/cgi-bin/RNAi_find_primers.pl), two independent gene segments for synthesis of *org-1* double-stranded RNA (dsRNA) were selected with lengths of 570 and 473 bp and with less than 20 and 22 bp of identity to any other predicted gene, respectively. These segments of the *org-1* gene were PCR-amplified from primary embryonic cDNA using the primers, CGTCCAAAAAGTTCAAGGGA and GCTCGTTCTCATCCAAGGAG (570 bp) and GCTCCAACAGAGCCAGAATC and CCGAACCGTAAAAACTTGGA (473 bp), and transcribed *in vitro* using the MEGAscript RNAi kit (Ambion, USA). *lbl-GFP*, *slou-GFP* or *MHC-tauGFP* embryos were dechorionated and injected with negative control (*lacZ*) or *org-1* dsRNA at the syncytial blastoderm stage and allowed to develop to stage 14 or 16 before examination by fluorescence microscopy for assessment of reporter GFP expression. A similar protocol was used to assess the function of *acj6* in *Ndg-GFP* embryos.

### FC Enhancer and Control Sequences

The sixteen sequences in the training set of *Drosophila melanogaster* FC enhancers range in length from 311 to 2068 bp (average length 1232 bp), in GC-content from 39% to 49% (average GC-content 43%), and in repeat-content from 0% to 7% (average 1.5%). The twenty-four orthologs have similar characteristics, with an average length of 1311 bp, GC-content of 43% and repeat content of 5%. The control set comprised 1000 randomly selected *D. melanogaster* noncoding genomic sequences with length, GC- and repeat-content matching those of the enhancer set.

### Identification of TFBSs

Putative TFBSs were identified by searching the sequences with MAST [118] for motifs in TRANSFAC Release 2009.2 [119], in addition to binding sequences for Tin, Twi and Pnt from the literature [5]. MAST was run independently on each individual sequence with default setup and parameters. In particular, for the final analysis of the TFBS composition of FC enhancers, we examined the sequences for the occurrence of 11 types of TFBSs: POUHD (V$OCT_01, V$POU1F1_Q6, V$OCT4_02), Tbx (V$TBX5_01, I$BYN_Q6), Myb (V$MYB_Q6), Fkh (V$FOXO3_01, V$FOXO1_Q5, V$FREAC2_01), HD (I$ABDA_Q6, V$CDX5_Q5, V$IFP_03, V$PAX4_02), and Mef2 (V$AMEF2_Q6, V$HMEF2_Q6) and binding sites for Tcf, Mad, Ets, Twi, Tin [5]. Since the Position Weight Matrices (PWMs) for Tcf, Mad, Ets, Twi, and Tin were constructed from only a small number of sequences and we obtained few significant matches using MAST, we re-inspected the sequences manually, searching for the known binding sites of these TFs.

Over-represented TFBSs were determined by comparing the occurrence of the motifs among query sequences and background genomic sequence, and applying Fisher's exact test. We used a P-value threshold of 0.05. When indicated, we adjusted the P-values for multiple testing using the procedure suggested by Benjamini and Hochberg [120].

### Classifier Training

Each nucleotide sequence in the FC enhancer and control sets was represented by the number of putative TFBSs per base pair. Putative TFBSs were identified by searching the sequences for motifs derived from three different sources: (1) 892 TF binding specificities characterized in TRANSFAC, (2) 3 binding sequences for Tin, Twi, and Pnt from the literature [5], and (3) at most 50 motifs that PRIORITY [51] found to be over-represented in the training set of FC enhancers. In order to prevent the over-representation of motifs in *D. melanogaster* enhancer sequences with a large number of orthologs, *de novo* motifs were identified on a restricted set of 34 sequences, including at most two randomly selected orthologs for each *D. melanogaster* FC enhancer. Features relevant for distinguishing between enhancer sequences and controls were identified using linear support vector machine (SVM). We used a standard ten-fold cross-validation procedure to assess the accuracy of the classifier. In each fold of the cross-validation procedure, the *de novo* motifs were extracted using the training data, only, thereby ensuring that the test data were completely unseen before the predictions were made. The cross-validation procedure should help to prevent overfitting of the classifier.

### SVM Parameter Selection

We used Support Vector Machines [121] with a linear kernel, which only requires setting the penalization coefficient C. The performance of the SVM was evaluated using the area under the receiver operating characteristic (ROC) curve, which yields values between 0.5 (for a completely random guess) and 1.0 (for a perfect classifier). In order to compensate for the data unbalance, FC enhancer sequences and controls were assigned different misclassification costs (SVM soft-margin constants), giving equal overall weight to each class.

### Motif Ranking

Given a training set of instances *x_1_, …, x_l_* 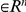 with associated labels *y_1_, …, y_l_* 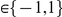, linear SVM solves the optimization problem 
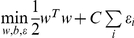
 subject to 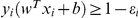 and 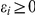 [122]. Thus, after obtaining a linear SVM model, the weight vector *w* can be used to decide the relevance of each feature [123]. The larger 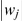, the more important role of feature j in the decision function. We rank features—in our case, motifs—according to 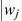. For this purpose, we trained a classifier for 100 random partitions of the training data (containing two thirds of the total training data), computed the ranking for each feature, and finally ranked the features according to their median ranking.

### Genome Scan

We scanned the whole-genome of *D. melanogaster* (BDGP Release 5 assembly) with a sliding window of length 1000 base pairs and overlaps of 500 base pairs. The length of the window corresponds approximately to the average length of the sequences in the training data set (1280 base pairs). We scored 137,364 sequences after excluding sequences which overlap annotated coding regions by at least 50%. The cut-off for the genome scan was defined to obtain a false positive rate (FPR) of at most 5% by training and testing 100 classifiers on random partitions of the training data (containing two thirds of the total training data).

### Association between TFs and Sequence Motifs

TF annotation for PWMs was obtained from TRANSFAC and the Broad Institute MsigDB database [124].

### Classifier Performance with Orthologs of *D. melanogaster* Mesodermal Enhancers

To understand the effects of a sample size on the classifier performances, we first extracted a dataset of *Drosophila melanogaster* 62 enhancers active in mesoderm from the REDfly database [32]. REDfly contains 176 partially overlapping enhancers active in mesoderm. To eliminate this redundancy, we clustered together overlapping sequences, and subsequently selected the shortest enhancer sequence from each cluster; sequences longer than 2 kb as well as sequences overlapping our dataset of enhancers active in muscle founder were excluded from the final dataset. Orthologs were selected randomly among sequences from 15 insect species [125] with nucleotide identity ranging from 50 to 80%, so that at most two orthologs were selected for each *Drosophila melanogaster* enhancer in REDfly. As controls, we randomly sampled for each enhancer (and orthologs) 10 non-coding sequences from the *Drosophila melanogaster* genome with similar length, GC- and repeat-content.

Each nucleotide sequence in the enhancer and control sets was represented by the number of putative TFBSs per base pair. Putative TFBSs were identified by searching the sequences with MAST [118] for motifs derived from two sources: (1) 892 TF binding specificities characterized in TRANSFAC Release 2009.2 [119], and (2) 3 binding sequences for Tin, Twi, and Pnt from the literature [5]. Features relevant for distinguishing between enhancer sequences and controls were identified using linear SVM.

## Supporting Information

Figure S1 Empirical validation of predicted FC enhancers conforming to a previously described regulatory model. Fluorescent *in situ* hybridization analysis of stage 11 embryos containing *RhoGEF3-lacZ* (A), *FBX011-lacZ* (B), *cib-lacZ* (C) or *fra-lacZ* (D) transgenes using probes for endogenous *RhoGEF3* (A), *FBX011* (B), *cib* (C), and *fra* (D) transcripts. Panels A′ to D′ show the corresponding signals for *lacZ* transcripts, and panels A″ to D″ show the merged channels. All enhancers were selected from previously identified candidates [5].
(TIF)

Figure S2 Variation of the classification performance with increasing sample size. Samples were randomly selected from a dataset of 62 *D. melanogaster* enhancers active in various mesodermal cell types. The sample size was varied from 10 to 60 by an increment of 5. Each sample was used to train a Support Vector Machine (SVM) classifier. For each sample size, we compared the performance of the classifier trained exclusively with *D. melanogaster* (“dm3 only”) enhancers with that of a classifier that, in addition, was trained with up to two orthologs for each *D. melanogaster* enhancer (“dm3 with orthologs”). All classifiers were validated on sets comprising only *D. melanogaster* enhancers. Control sequences were randomly selected from regions of the *D. melanogaster* genome with comparable length, GC- and repeat-content. The entire process was repeated a total of 1000 times. (A) Performance of each classifier, measured by its AUC, estimated in a 10-fold cross-validation. Classifiers trained on *D. melanogaster* enhancers and their orthologous sequences with an AUC significantly greater than that of the corresponding classifier trained exclusively on *D. melanogaster* enhancers (P<0.05, Wilcoxon sign rank test) are marked with a red asterisk. (B) Precision of the estimated calculated based on the Root Mean Square (RMS) error. The RMS describes how well the AUC value estimated in the cross-validation represents the true AUC of the classifier and thus, how good is our assessment of the underlying model; the true AUC of each classifier was computed using the enhancers excluded from the randomly selected sample. (C) Number of enhancers recognized as such in at least 50% of the instances in which they were tested. In the cross-validation process each sequence is used exactly once for validation. Thus, for 100 randomly selected samples and their corresponding cross-validation processes, we counted the number of times each sequence scored positively, compared this number with the number of times each sequence had been included in a random sample, and repeated the complete procedure 10 times to estimate the variance of the results. Classifiers trained on *D. melanogaster* enhancers and their orthologous sequences consistently recognizing a significantly higher number of sequences as compared with the respective classifiers trained only on *D. melanogaster* enhancers (P<0.05, Wilcoxon sign rank test) are marked with a red asterisk. (D) Concordance of prediction outcome between each pair of 1000 classifiers, for each sample size. In this graph, the line segments represent the 95% confidence intervals surrounding the means. We compared the sequences positively scoring in the 10 folds of the cross-validation experiment between each pair of classifiers. Randomly sampled training and test datasets differ. The likelihood of observing a large overlap between two samples taken from the same (finite) population increases with the size of the samples. Therefore, larger datasets produce larger overlapping outcomes. However, for the same sample size, the overlap between the outcomes of classifiers trained on *D. melanogaster* enhancers and their orthologous sequences is systematically significantly higher as compared to that of classifiers trained only on *D. melanogaster*.
(TIF)

Figure S3 Binding site conservation and evolutionary flux in orthologous FC enhancers. Sequences of the *lbl* (A), ap (B), *Ndg* (C) and eve (D) enhancers were aligned against the orthologous enhancers of *D. persimilis* (*D. per*), *D. ananassae* (*D. ana*), or *D. mojavensis (D. moj)*. Motif matches to Tcf (black), Mad (blue), Ets (red), Twi (green) and Tin (purple) for these co-regulating TFs of the *eve* MHE are shown. For the *Ndg*, lbl, and ap enhancers, motif matches to the co-regulating TFs Ets (red), Twi (green) and Tin (purple) are shown. Motifs are based on known functional binding sites (*D. mel eve* MHE, [28]) or matrices compiled from the literature for Tcf, Mad, Twi and Tin [5] for the other sequences. K-mer matches for protein binding microarray data for the mouse ortholog of Pnt (Ets1) are shown for Ets binding sites. For *lbl*, a motif match to a functional Slou-preferred binding site (blue) is shown (B. W. Busser, L. Shokri, S. A. Jaeger, S. S. Gisselbrecht, A. Singhania, M. F. Berger, B. Zhou, M. L. Bulyk and A. M. Michelson, unpublished data). For *ap*, Antennapedia (Antp)-protected functional binding sites are shown for *D. melanogaster* [35]. Similar sites predicted with protein binding microarray data for Ubx and AbdB are shown for *D. mojavensis* ([35] and B. W. Busser, L. Shokri, S. A. Jaeger, S. S. Gisselbrecht, A. Singhania, M. F. Berger, B. Zhou, M. L. Bulyk and A. M. Michelson, unpublished data).
(TIF)

Figure S4 TFs most relevant to the FC enhancer classification. Presence (red)/absence (gray) of the fifty most relevant TF binding motifs in the set of enhancer sequences used for training. TFs were ranked according to the SVM weights of their respective motifs, which represent their discriminating power. We only considered the highest scoring motif for each TF (median ranks computed across 10 random partitions of the training data varied between 12 and 129). Control TFs were randomly chosen among TFs for which the highest scoring motif had a neutral weight (median ranks computed across 10 random partitions of the training data varied between 437 and 450). TFs and sequences have been clustered using average linkage and Euclidean distance. The phylogenetic tree represents the relations among the sequences in the training data, built on the presence/absence of the motifs for the most relevant TFs. De novo motifs were explicitly excluded from this analysis.
(TIF)

Figure S5 PRIORITY motifs are representations of known motifs. Many of the *de novo* motifs exhibiting the highest power discriminating FC enhancers from background sequence resemble motifs of known transcription factors with roles in mesoderm and FC development. The identity of the transcription factors binding to the *de novo* motifs was queried using STAMP [60] and the data set of binding affinities FlyReg [32].
(TIF)

Figure S6 Motifs identified by the classifier that are overrepresented in FC enhancers and their orthologs. Graphs comparing the representation of V$ETS_Q4 (A), V$POU1F1_Q6 (B), V$MYB_Q6 (C) V$TBX5_01 (D) and V$FOX03_01 (E) motifs in *D. melanogaster* FC enhancers (dm3) and orthologous and control sequences. Position weight matrices for each of these TFs are also shown.
(TIF)

Figure S7 The wild-type activities of FC enhancers require input from classifier-defined Ets and Fkh TF binding motifs. (A) GFP (green) expression driven by a version of the *Ndg* enhancer in which Ets sites are selectively inactivated (*Ndg^ETS^-GFP*) is extinguished compared to β-Gal (magenta) driven by *Ndg^WT^-lacZ*. We have previously demonstrated the activity of this enhancer in a subset of FCs, two pericardial and two cardial cells of the heart, the gut musculature and two cells of the central nervous system ([5] and X. Zhu, S. M. Ahmad, A. Aboukhalil, B. W. Busser, Y. Kim, T. R. Tansey, A. Haimovich, N. Jeffries, M. L. Bulyk, and A. M. Michelson, unpublished data). Of note, the entirety of this expression pattern is extinguished in the absence of Ets binding sites, while the reporter is de-repressed into additional cells of the central nervous system (Figure S7A and data not shown). The locations of Ets binding sites in the *Ndg* enhancer are indicated in Figure 6B and Table S4. (B) TRANSFAC position weight matrix for the Fkh (V$FOX03_01) enriched motif identified by the classifier, and locations of Fkh binding sites in the *ap* muscle FC enhancer. Although the *Ndg* enhancer contains several examples of this motif, mutagenesis studies revealed that Fkh binding sites are not required for the expression of *Ndg* in muscle FCs (X. Zhu, S. M. Ahmad, A. Aboukhalil, B. W. Busser, Y. Kim, T. R. Tansey, A. Haimovich, N. Jeffries, M. L. Bulyk, and A. M. Michelson, unpublished data). (C) Activity of the wild-type *ap* enhancer in lateral transverse muscles, as revealed by GFP expression driven by the *ap^WT^-GFP* transgene. (D) Complete loss of *ap* enhancer activity after Fkh binding sites are inactivated (*ap^Fkh^-GFP*).
(TIF)

Figure S8 Co-expression of *org-1* with Slou and Lbl. Expression of *org-1* RNA in stage 11 (A) and stage 13 (B) embryos detected by *in situ* hybridization. Co-expression of *org-1* RNA (purple) with Lbl protein (brown) in the Lbl-expressing SBM FC (stage 11; C) and myotube (stage 13; D). Co-expression of *org-1* RNA (purple) with Slou protein (brown) in the LO1 and VT1 FCs (stage 11; E) and myotubes (stage 13; F). CVM: circular visceral muscle which expresses *org-1* but neither *lbl* nor *slou*.
(TIF)

Figure S9 Motif distribution in FC enhancers. (A) The heatmap illustrates the occurrence of 11 motifs that have been shown to be relevant for FC regulation in the 18 sequences that have been positively assayed for FC enhancer activity. Columns and rows are clustered using Ward's method and binary distances. (B) Maximum fraction of the 18 assayed FC enhancer sequences sharing N motifs that have been shown to be relevant for FC regulation, for N in {1, 2, …, 11}.
(TIF)

Table S1 Expression and genomic coordinates of *D. melanogaster* and orthologous enhancer regions used for training, and the list of FC genes considered in this study.
(XLSX)

Table S2 Genomic coordinates and ontology of REDfly *D. melanogaster* mesodermal enhancers. The majority of these enhancers have multiple activities.
(XLSX)

Table S3 Classifier predictions and the activity and genomic coordinates of the tested enhancer predictions.
(XLSX)

Table S4 Motifs identified by the classifier and the mapping of TFBSs in the *Ndg*, *lbl*, *slou* and *ap* FC enhancers.
(XLSX)

Table S5 Summary of *in situ* hybridization analysis of T-box and POUHD family members.
(XLSX)

Table S6 Mapping of TFBSs in all FC enhancers.
(XLSX)

Text S1 Conservation Profile of Candidate FC Enhancers and TFBS Distribution Among Orthologs of Candidate FC Enhancers.
(DOCX)
