# Supplementary figures and images for: A Machine Learning Approach for Identifying Novel Cell Type–Specific Transcriptional Regulators of Myogenesis
Source: PLoS Genet. 2012 Mar 8;8(3):e1002531. doi: 10.1371/journal.pgen.1002531 (PMC3297574; doi:10.1371/journal.pgen.1002531)

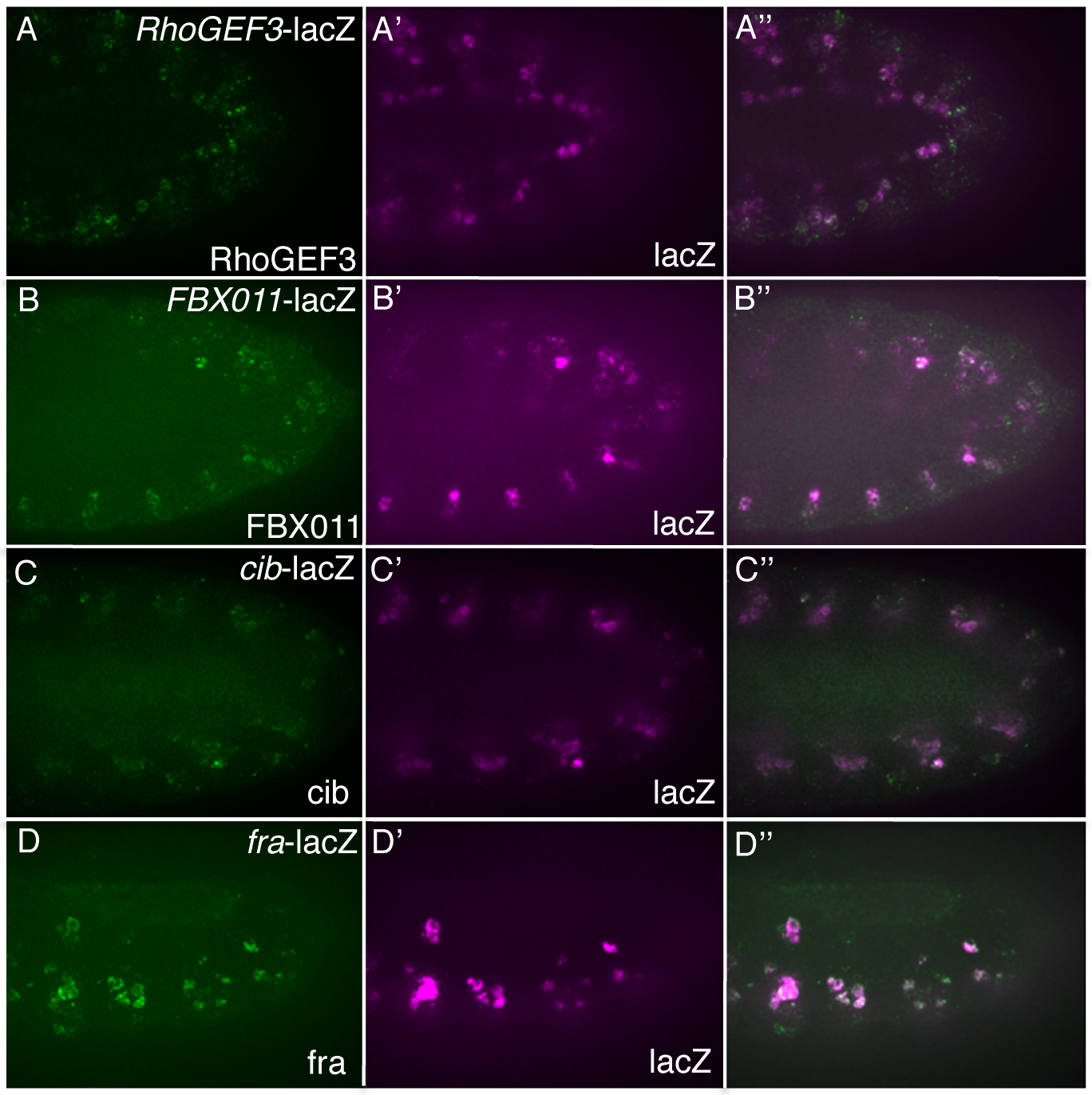

Supplement: Figure S1 — Empirical validation of predicted FC enhancers conforming to a previously described regulatory model. Fluorescent in situ hybridization analysis of stage 11 embryos containing RhoGEF3-lacZ (A), FBX011-lacZ (B), cib-lacZ (C) or fra-lacZ (D) transgenes using probes for endogenous RhoGEF3 (A), FBX011 (B), cib (C), and fra (D) transcripts. Panels A′ to D′ show the corresponding signals for lacZ transcripts, and panels A″ to D″ show the merged channels. All enhancers were selected from previously identified candidates [5]. (TIF) [file pgen.1002531.s001.tif]

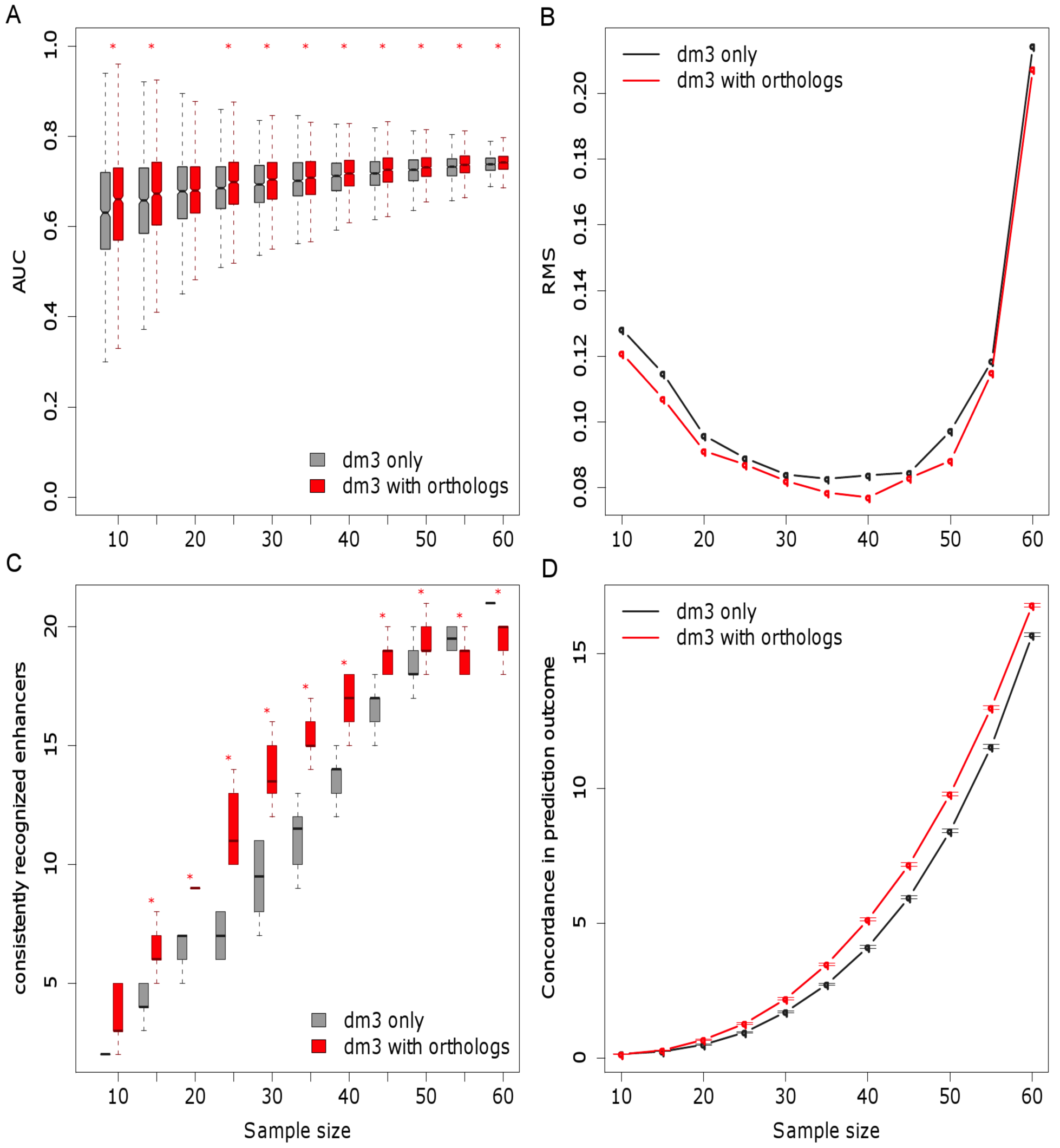

Supplement: Figure S2 — Variation of the classification performance with increasing sample size. Samples were randomly selected from a dataset of 62 D. melanogaster enhancers active in various mesodermal cell types. The sample size was varied from 10 to 60 by an increment of 5. Each sample was used to train a Support Vector Machine (SVM) classifier. For each sample size, we compared the performance of the classifier trained exclusively with D. melanogaster (“dm3 only”) enhancers with that of a classifier that, in addition, was trained with up to two orthologs for each D. melanogaster enhancer (“dm3 with orthologs”). All classifiers were validated on sets comprising only D. melanogaster enhancers. Control sequences were randomly selected from regions of the D. melanogaster genome with comparable length, GC- and repeat-content. The entire process was repeated a total of 1000 times. (A) Performance of each classifier, measured by its AUC, estimated in a 10-fold cross-validation. Classifiers trained on D. melanogaster enhancers and their orthologous sequences with an AUC significantly greater than that of the corresponding classifier trained exclusively on D. melanogaster enhancers (P<0.05, Wilcoxon sign rank test) are marked with a red asterisk. (B) Precision of the estimated calculated based on the Root Mean Square (RMS) error. The RMS describes how well the AUC value estimated in the cross-validation represents the true AUC of the classifier and thus, how good is our assessment of the underlying model; the true AUC of each classifier was computed using the enhancers excluded from the randomly selected sample. (C) Number of enhancers recognized as such in at least 50% of the instances in which they were tested. In the cross-validation process each sequence is used exactly once for validation. Thus, for 100 randomly selected samples and their corresponding cross-validation processes, we counted the number of times each sequence scored positively, compared this number with the number of times [file pgen.1002531.s002.tif]

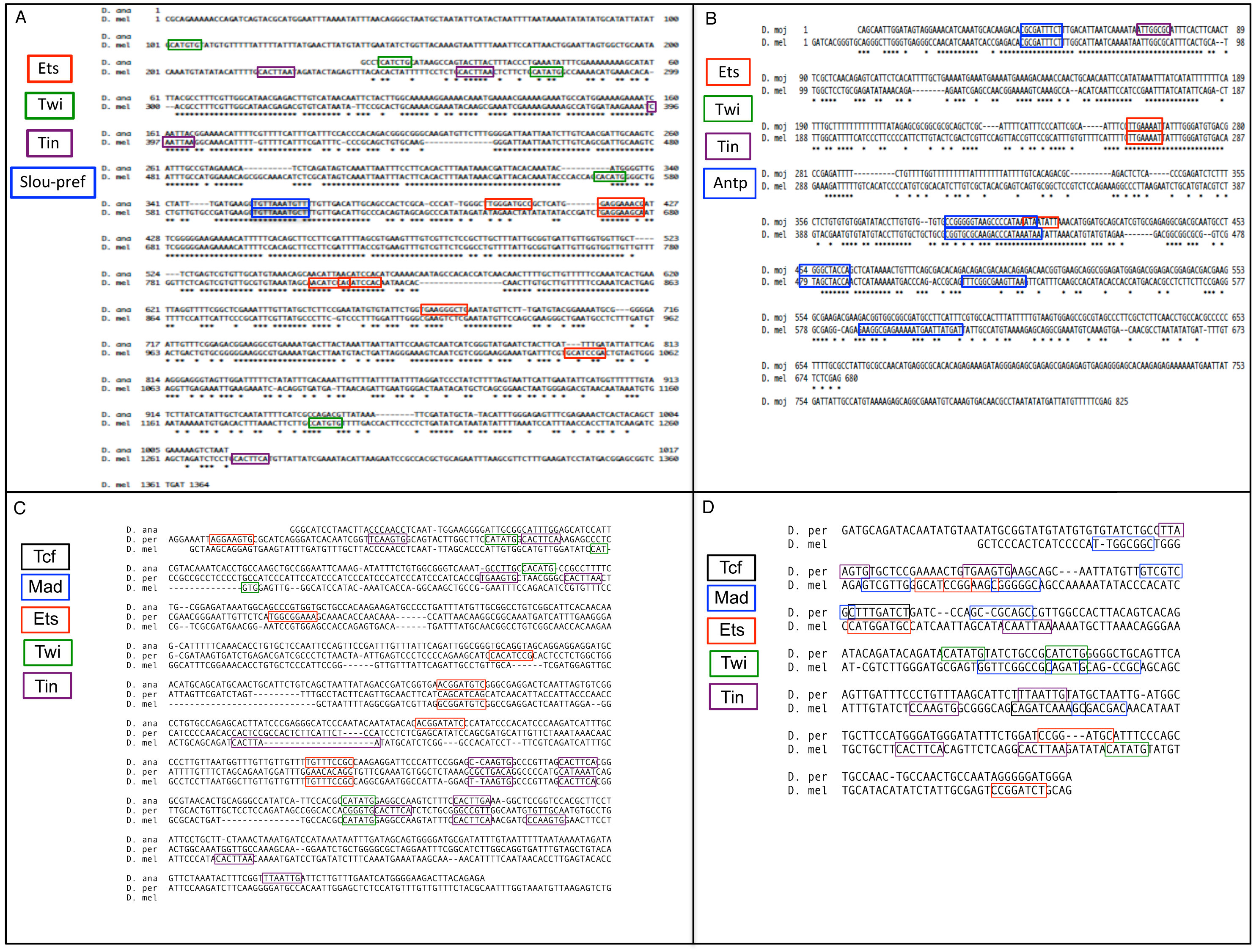

Supplement: Figure S3 — Binding site conservation and evolutionary flux in orthologous FC enhancers. Sequences of the lbl (A), ap (B), Ndg (C) and eve (D) enhancers were aligned against the orthologous enhancers of D. persimilis (D. per), D. ananassae (D. ana), or D. mojavensis (D. moj). Motif matches to Tcf (black), Mad (blue), Ets (red), Twi (green) and Tin (purple) for these co-regulating TFs of the eve MHE are shown. For the Ndg, lbl, and ap enhancers, motif matches to the co-regulating TFs Ets (red), Twi (green) and Tin (purple) are shown. Motifs are based on known functional binding sites (D. mel eve MHE, [28]) or matrices compiled from the literature for Tcf, Mad, Twi and Tin [5] for the other sequences. K-mer matches for protein binding microarray data for the mouse ortholog of Pnt (Ets1) are shown for Ets binding sites. For lbl, a motif match to a functional Slou-preferred binding site (blue) is shown (B. W. Busser, L. Shokri, S. A. Jaeger, S. S. Gisselbrecht, A. Singhania, M. F. Berger, B. Zhou, M. L. Bulyk and A. M. Michelson, unpublished data). For ap, Antennapedia (Antp)-protected functional binding sites are shown for D. melanogaster [35]. Similar sites predicted with protein binding microarray data for Ubx and AbdB are shown for D. mojavensis ([35] and B. W. Busser, L. Shokri, S. A. Jaeger, S. S. Gisselbrecht, A. Singhania, M. F. Berger, B. Zhou, M. L. Bulyk and A. M. Michelson, unpublished data). (TIF) [file pgen.1002531.s003.tif]

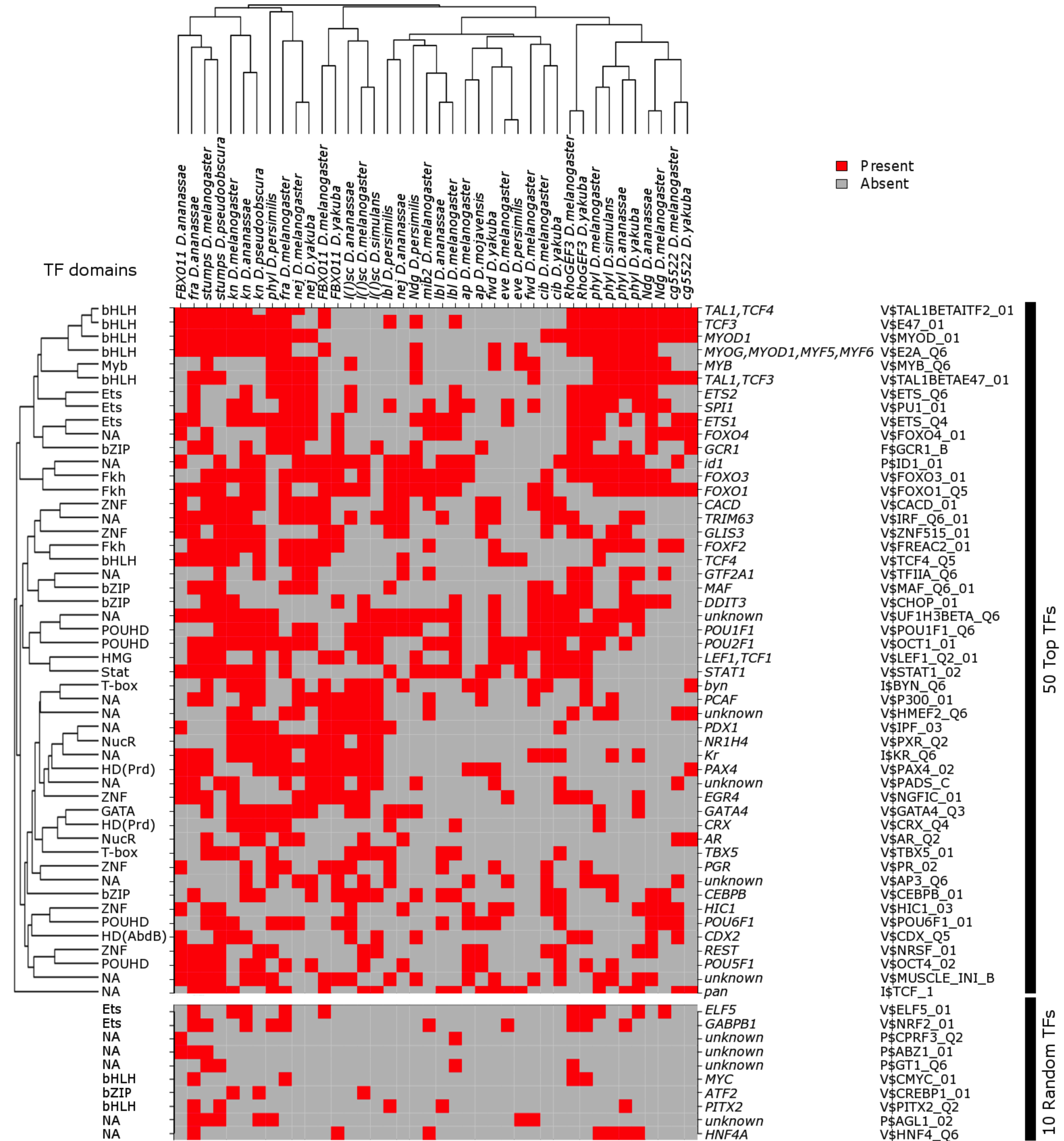

Supplement: Figure S4 — TFs most relevant to the FC enhancer classification. Presence (red)/absence (gray) of the fifty most relevant TF binding motifs in the set of enhancer sequences used for training. TFs were ranked according to the SVM weights of their respective motifs, which represent their discriminating power. We only considered the highest scoring motif for each TF (median ranks computed across 10 random partitions of the training data varied between 12 and 129). Control TFs were randomly chosen among TFs for which the highest scoring motif had a neutral weight (median ranks computed across 10 random partitions of the training data varied between 437 and 450). TFs and sequences have been clustered using average linkage and Euclidean distance. The phylogenetic tree represents the relations among the sequences in the training data, built on the presence/absence of the motifs for the most relevant TFs. De novo motifs were explicitly excluded from this analysis. (TIF) [file pgen.1002531.s004.tif]

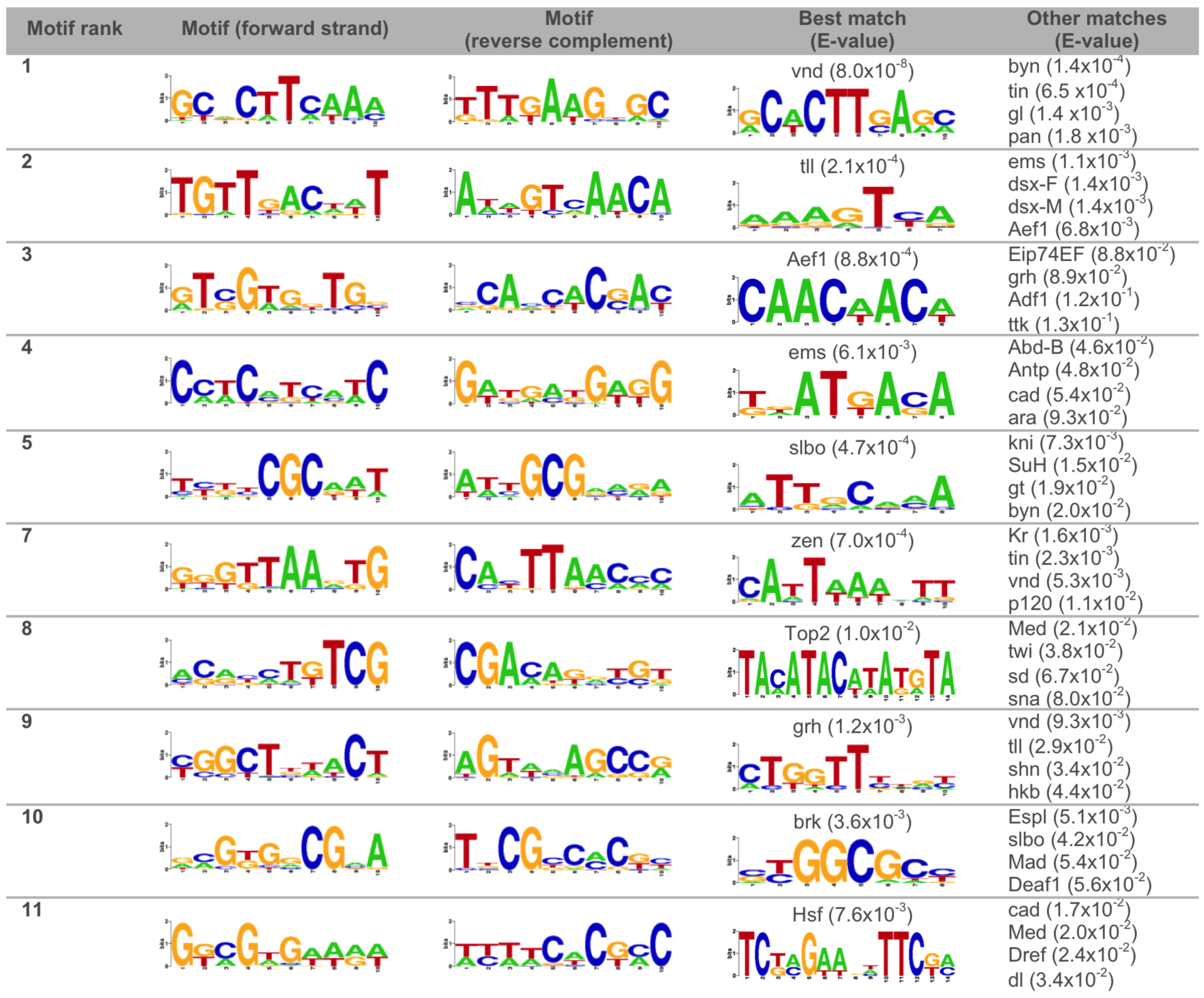

Supplement: Figure S5 — PRIORITY motifs are representations of known motifs. Many of the de novo motifs exhibiting the highest power discriminating FC enhancers from background sequence resemble motifs of known transcription factors with roles in mesoderm and FC development. The identity of the transcription factors binding to the de novo motifs was queried using STAMP [60] and the data set of binding affinities FlyReg [32]. (TIF) [file pgen.1002531.s005.tif]

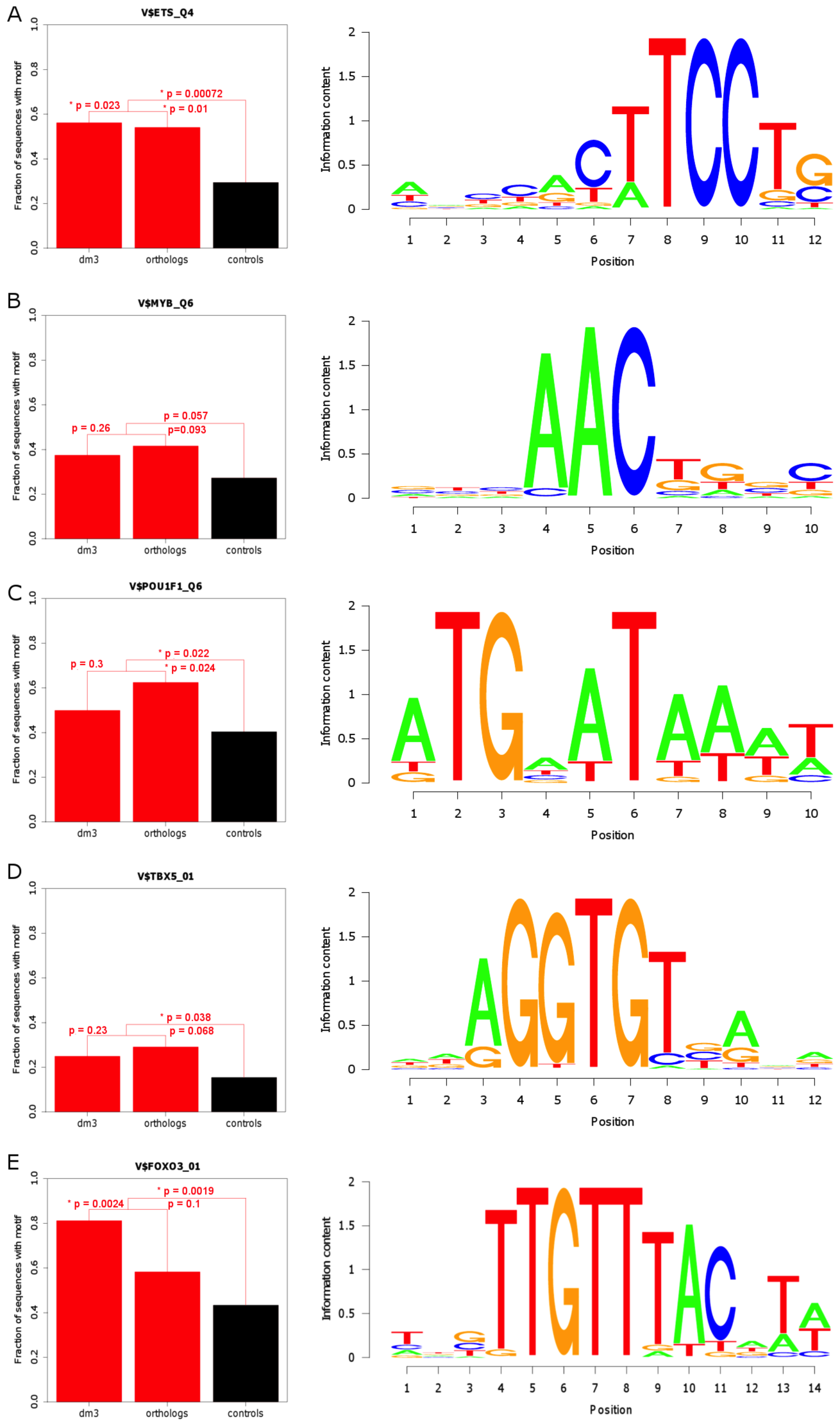

Supplement: Figure S6 — Motifs identified by the classifier that are overrepresented in FC enhancers and their orthologs. Graphs comparing the representation of V$ETS_Q4 (A), V$POU1F1_Q6 (B), V$MYB_Q6 (C) V$TBX5_01 (D) and V$FOX03_01 (E) motifs in D. melanogaster FC enhancers (dm3) and orthologous and control sequences. Position weight matrices for each of these TFs are also shown. (TIF) [file pgen.1002531.s006.tif]

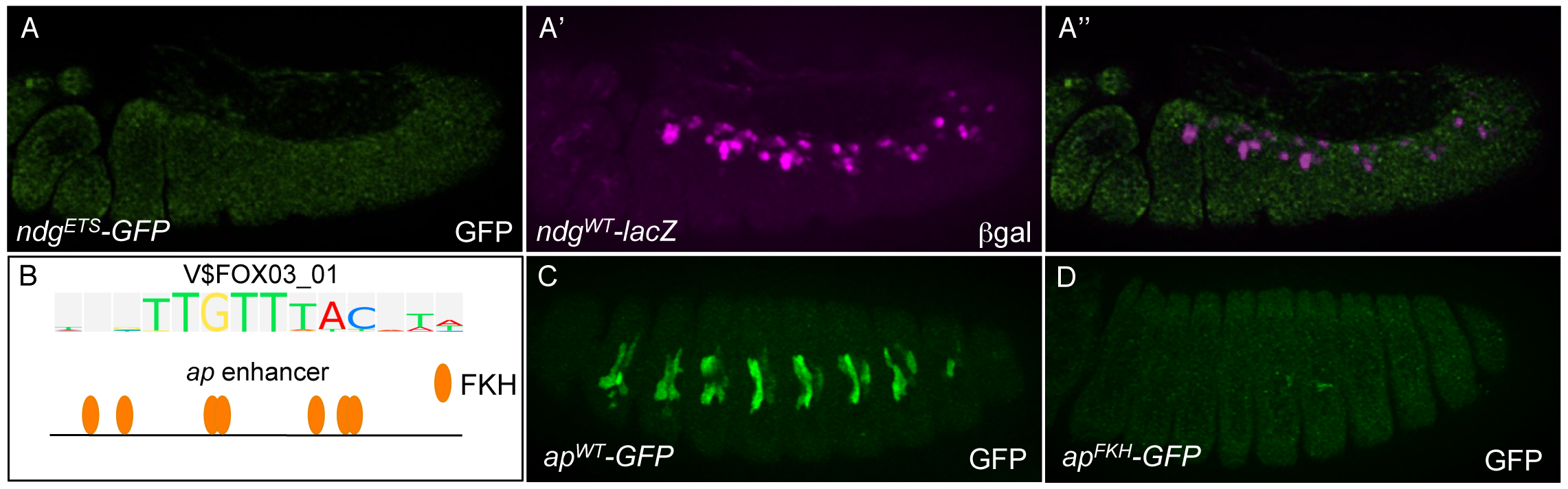

Supplement: Figure S7 — The wild-type activities of FC enhancers require input from classifier-defined Ets and Fkh TF binding motifs. (A) GFP (green) expression driven by a version of the Ndg enhancer in which Ets sites are selectively inactivated (NdgETS-GFP) is extinguished compared to β-Gal (magenta) driven by NdgWT-lacZ. We have previously demonstrated the activity of this enhancer in a subset of FCs, two pericardial and two cardial cells of the heart, the gut musculature and two cells of the central nervous system ([5] and X. Zhu, S. M. Ahmad, A. Aboukhalil, B. W. Busser, Y. Kim, T. R. Tansey, A. Haimovich, N. Jeffries, M. L. Bulyk, and A. M. Michelson, unpublished data). Of note, the entirety of this expression pattern is extinguished in the absence of Ets binding sites, while the reporter is de-repressed into additional cells of the central nervous system (Figure S7A and data not shown). The locations of Ets binding sites in the Ndg enhancer are indicated in Figure 6B and Table S4. (B) TRANSFAC position weight matrix for the Fkh (V$FOX03_01) enriched motif identified by the classifier, and locations of Fkh binding sites in the ap muscle FC enhancer. Although the Ndg enhancer contains several examples of this motif, mutagenesis studies revealed that Fkh binding sites are not required for the expression of Ndg in muscle FCs (X. Zhu, S. M. Ahmad, A. Aboukhalil, B. W. Busser, Y. Kim, T. R. Tansey, A. Haimovich, N. Jeffries, M. L. Bulyk, and A. M. Michelson, unpublished data). (C) Activity of the wild-type ap enhancer in lateral transverse muscles, as revealed by GFP expression driven by the apWT-GFP transgene. (D) Complete loss of ap enhancer activity after Fkh binding sites are inactivated (apFkh-GFP). (TIF) [file pgen.1002531.s007.tif]

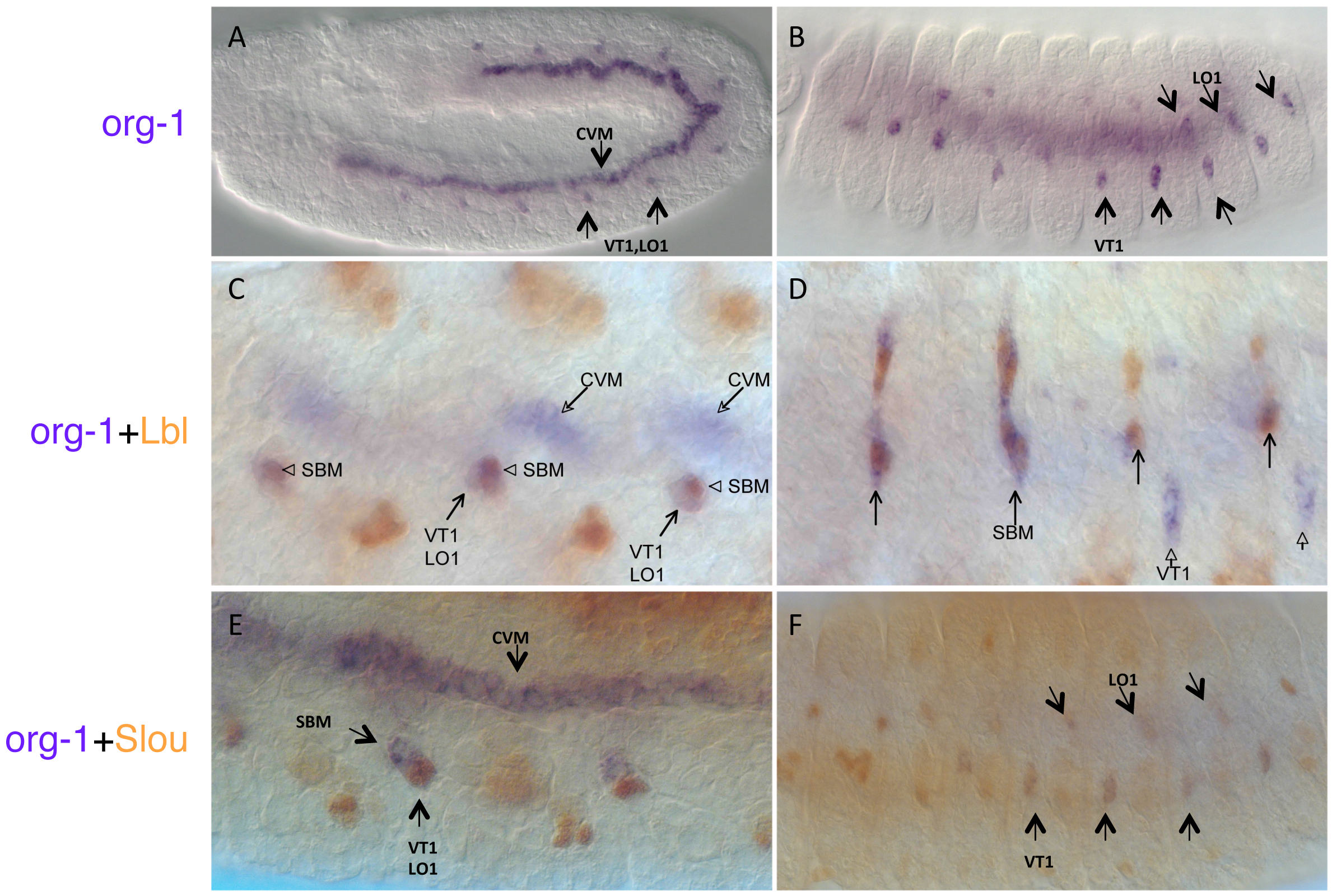

Supplement: Figure S8 — Co-expression of org-1 with Slou and Lbl. Expression of org-1 RNA in stage 11 (A) and stage 13 (B) embryos detected by in situ hybridization. Co-expression of org-1 RNA (purple) with Lbl protein (brown) in the Lbl-expressing SBM FC (stage 11; C) and myotube (stage 13; D). Co-expression of org-1 RNA (purple) with Slou protein (brown) in the LO1 and VT1 FCs (stage 11; E) and myotubes (stage 13; F). CVM: circular visceral muscle which expresses org-1 but neither lbl nor slou. (TIF) [file pgen.1002531.s008.tif]

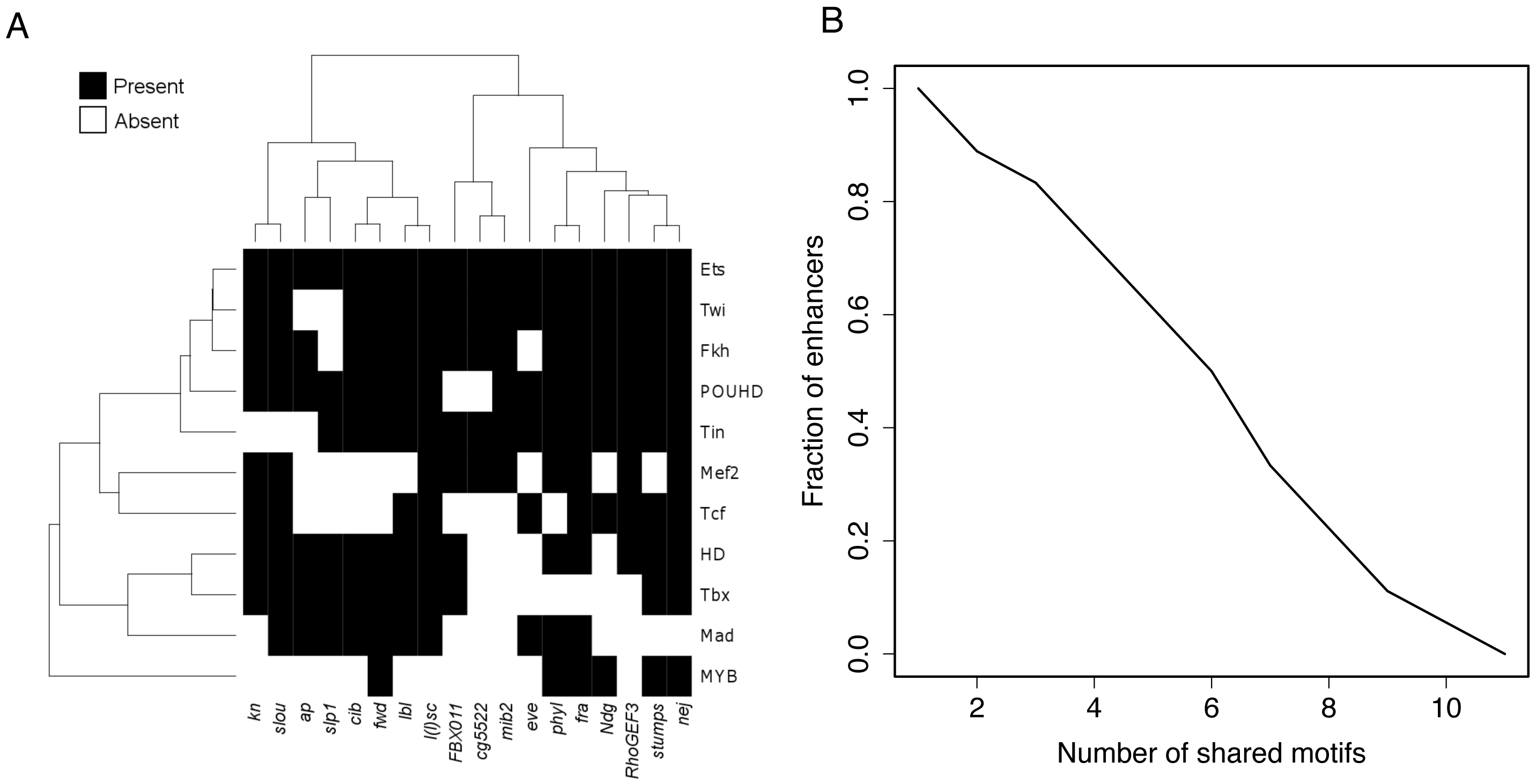

Supplement: Figure S9 — Motif distribution in FC enhancers. (A) The heatmap illustrates the occurrence of 11 motifs that have been shown to be relevant for FC regulation in the 18 sequences that have been positively assayed for FC enhancer activity. Columns and rows are clustered using Ward's method and binary distances. (B) Maximum fraction of the 18 assayed FC enhancer sequences sharing N motifs that have been shown to be relevant for FC regulation, for N in {1, 2, …, 11}. (TIF) [file pgen.1002531.s009.tif]
