# Supplementary material for: A Machine Learning Approach for Identifying Novel Cell Type–Specific Transcriptional Regulators of Myogenesis
Source: PLoS Genet. 2012 Mar 8;8(3):e1002531. doi: 10.1371/journal.pgen.1002531 (PMC3297574; doi:10.1371/journal.pgen.1002531)
Supplement: Text S1 — Conservation Profile of Candidate FC Enhancers and TFBS Distribution Among Orthologs of Candidate FC Enhancers. (DOCX) [file pgen.1002531.s016.docx]

**A machine learning approach for identifying novel cell type-specific transcriptional regulators of myogenesis**

Brian W. Busser^1*^, Leila Taher^2*^, Yongsok Kim^1^, Terese Tansey^1^, Molly J. Bloom^1^,

Ivan Ovcharenko^2†^ and Alan M. Michelson^1†^

^1^Laboratory of Developmental Systems Biology, Genetics and Developmental Biology Center, Division of Intramural Research, National Heart Lung and Blood Institute, National Institutes of Health, Bethesda, MD 20892, USA.

^2^Computational Biology Branch, National Center for Biotechnology Information, National Library of Medicine, National Institutes of Health, Bethesda, MD 20892, USA.

^*^These authors contributed equally to this work.

^†^To whom correspondence should be addressed. Email: [ovcharen@nih.gov](mailto:ovcharen@nih.gov) (I.O.); [michelsonam@nhlbi.nih.gov](mailto:michelsonam@nhlbi.nih.gov) (A.M.M.)**.**

**Text S1: Supplemental Materials**

**Conservation Profile of Candidate FC Enhancers**

Comparative genomics analysis of *D. melanogaster* sequences identified as putative FC enhancers in 14 species, including 11 *Drosophila* species, mosquito, honeybee, and red flour beetle, reveals their overall conservation profile (Figure 1). As expected, non-coding sequence conservation diminishes steadily with increasing evolutionary distance from *D. melanogaster*. *D. simulans* and *D. sechellia* are the most closely related species to *D. melanogaster*, and therefore, orthologs of *D. melanogaster* candidate FC enhancers are very well conserved in these species, and over 80% of them are within the 80-100% range of sequence identity. On the other hand, over 20% of orthologs in the more distant *D. yakuba* and *D. erecta*, and over 50% in the even more distant *D. ananassae, D. pseudoobscura* and *D. persimillis*, are within the 50-80% range of identity, making these sequences good candidates for our phylogenetic profiling strategy. Finally, sequences in further related species are only very weakly conserved, if at all. Rather than lineage-specific losses of putative FC enhancers, we see a gradual decrease in the overall percentage of sequence identity. Allowing for differences arising from the small sample size, this distribution of sequences in the 50-80% range of identity across the different species is consistent with the composition of our training set.

In addition, *D. melanogaster* candidate FC enhancers tend to be more deeply conserved than background genomic sequence, a result that is consistent with the observation that functionally relevant sequences are more constrained than non-functional sequences, and that is also evident in the training set. This effect is significant for all *Drosophila* species, except for more distantly related species, such as *D. virilis* and *D. grimshawi* (P-values < 0.05, computed using the Binomial test, corrected for multiple testing using Bonferroni’s method)*.* In particular, we do not observe substantial differences between the 12 sequences assayed for enhancer activity and the training set of *D. melanogaster* FC enhancers.

Figure 1. Sequence identity of *D. melanogaster* candidate regulatory sequences with different *Drosophila* species, mosquito, honeybee, and red flour beetle.

**TFBS Distribution among Orthologs of Candidate FC Enhancers**

Conservation of relevant binding sites in orthologs of *D. melanogaster* putative FC enhancers is correlated with sequence identity, and is often higher than the random expectation, in agreement with the proposed functionality of the sites (see Figure 2 for an example). Putative TFBSs were identified by searching the sequences with MAST ([Bailey and Elkan, 1994](#_ENREF_1)) for POUHD (V$OCT1_01, V$POU1F1_Q6, V$OCT4_02) Tbx (V$TBX5_01, I$BYN_Q6), MYB (V$MYB_Q6), Fkh (V$FOXO1_Q5, V$FOXO3_01, V$FREAC2_01), Mef2 (V$HMEF2_Q6, V$AMEF2_Q6), and HD (I$ABDA_Q6, V$CDX_Q5, V$IPF_03, V$PAX4_02) motifs in TRANSFAC Release 2009.2 ([Matys, et al., 2006](#_ENREF_3)), in addition to binding sequences for Tin, Twi, Pnt, Mad, and Tcf from the literature ([Philippakis, et al., 2006](#_ENREF_4)). MAST was run using its default setup and default parameters. To overcome the limitations of alignment-based methods to detect regulatory-motifs, we employed a relaxed definition of conservation, considering that a binding site is conserved if it occurs anywhere across the sequences compared ([Gordan, et al., 2010](#_ENREF_2); [Taher, et al., 2011](#_ENREF_5)). All these results are in agreement with functional sequences being generally more conserved than non-functionally related sequences, and within the normal range of expected variation.

Similarly, we investigated the conservation of the relative order among combinations of three binding sites (“triplets”) occurring in *D. melanogaster* and orthologous sequences with 50-80% identity across other *Drosophila* species, mosquito, honeybee and red flour beetle. We found an alteration in the relative order of the binding sites in approximately 25% of the triplets conserved across pairs of sequences, which does not differ from the background genomic expectation (Wilcoxon rank sum test, P-value > 0.05), suggesting a weak evolutionary constraint on the order of these binding sites.

We would like to emphasize that, since these results rely exclusively on computational predictions of individual transcription factor binding sites whose functions have not been independently validated they need to be treated with caution.


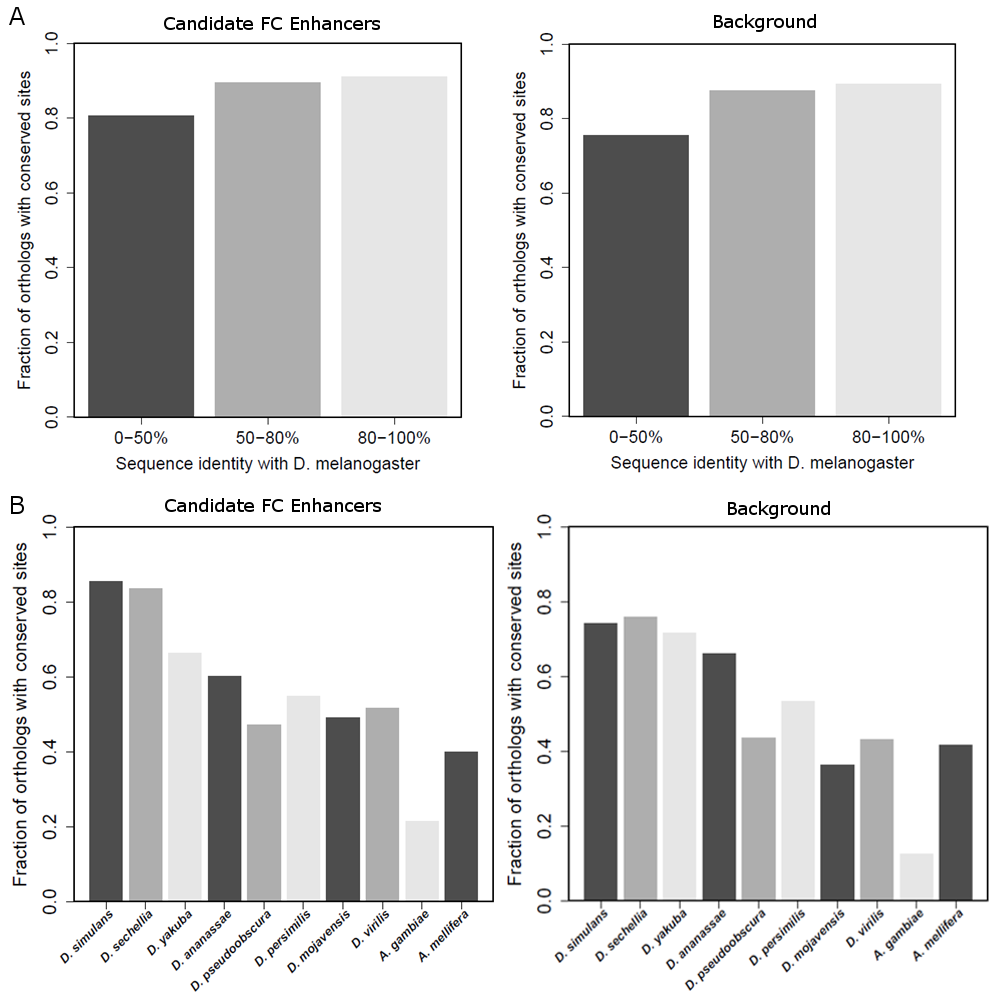


Figure 2. Conservation of binding sites for POU1F1 among orthologs of *D. melanogaster* in candidate FC enhancers and background genomic sequence as a function of a) sequence identity, and b) species sorted following their phylogeny.

**References**

Bailey, T.L. and Elkan, C. (1994) Fitting a mixture model by expectation maximization to discover motifs in biopolymers, *Proc Int Conf Intell Syst Mol Biol*, **2**, 28-36.

Gordan, R., Narlikar, L. and Hartemink, A.J. (2010) Finding regulatory DNA motifs using alignment-free evolutionary conservation information, *Nucleic Acids Res*, **38**, e90.

Matys, V.*, et al.* (2006) TRANSFAC and its module TRANSCompel: transcriptional gene regulation in eukaryotes, *Nucleic Acids Res*, **34**, D108-110.

Philippakis, A.A.*, et al.* (2006) Expression-guided in silico evaluation of candidate cis regulatory codes for Drosophila muscle founder cells, *PLoS Comput Biol*, **2**, e53.

Taher, L.*, et al.* (2011) Genome-wide identification of conserved regulatory function in diverged sequences, *Genome Res*, **21**, 1139-1149.
